# An Efficient, Optimized Synthesis of Fentanyl and Related Analogs

**DOI:** 10.1371/journal.pone.0108250

**Published:** 2014-09-18

**Authors:** Carlos A. Valdez, Roald N. Leif, Brian P. Mayer

**Affiliations:** 1 Physical and Life Sciences Directorate, Lawrence Livermore National Laboratory, Livermore, California, United States of America; 2 Forensic Science Center, Lawrence Livermore National Laboratory, Livermore, California, United States of America; Bangor University, United Kingdom

## Abstract

The alternate and optimized syntheses of the parent opioid fentanyl and its analogs are described. The routes presented exhibit high-yielding transformations leading to these powerful analgesics after optimization studies were carried out for each synthetic step. The general three-step strategy produced a panel of four fentanyls in excellent yields (73–78%) along with their more commonly encountered hydrochloride and citric acid salts. The following strategy offers the opportunity for the gram-scale, efficient production of this interesting class of opioid alkaloids.

## Introduction

**Figure 1:**
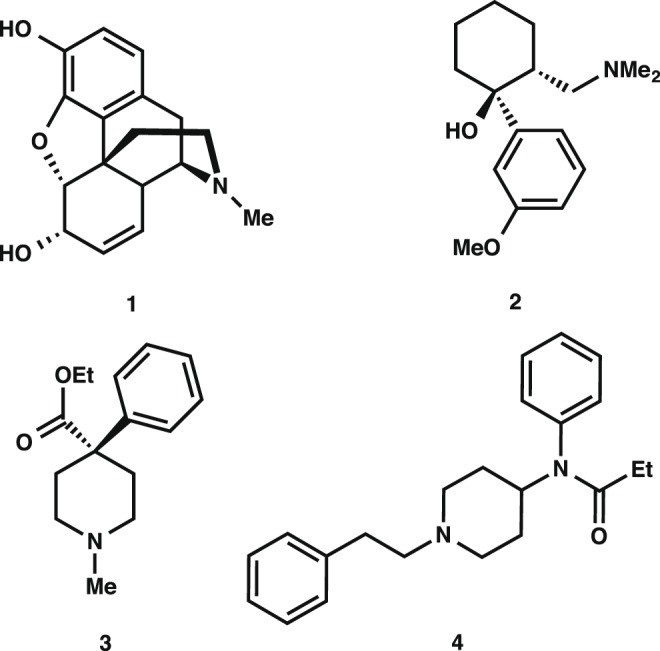
Various commonly administered opioids: (1) morphine, (2) Tramadol, (3) Demerol, and (4) fentanyl.

Very few synthetic drugs generate an immediate and powerful impact in the biomedical field shortly after their inception. This has been the case particularly within the areas of pre-surgical, surgical, and post-surgical anesthesiology where the need for fast acting, effective pain relievers is a key element in the overall patient care practice. Morphine (**1**) and Tramadol (**2**) (Fig. 1) are two opioid-based compounds that are widely recognized for being the gold standard prescriptions for patients with moderate to severe pain after surgery or with certain disease states (*e.g.* cancer) [1]–[6]. Due to their potency, these therapeutics, however, are also well known for their ability to foster chemical dependencies in patients and other users [7].

**Figure 2:**
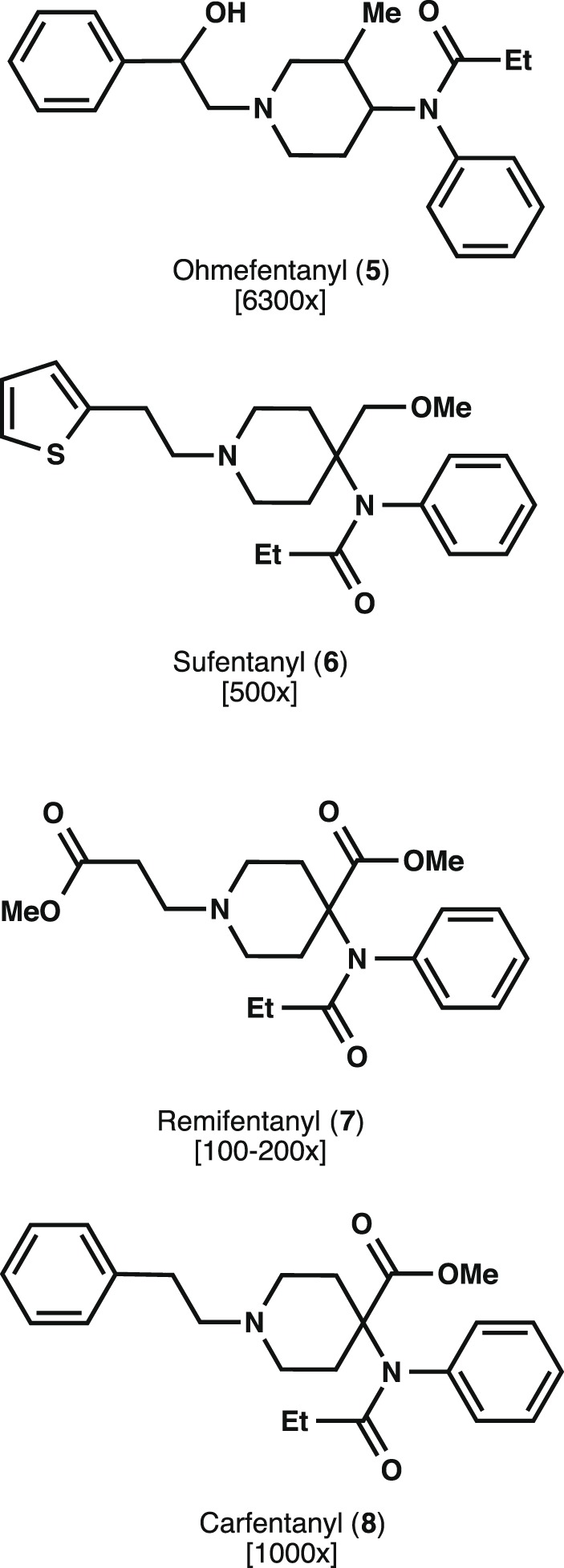
Fentanyl analogs and their potencies relative to morphine.

Though it is often difficult to surpass the established therapeutic records and efficiency profiles by the aforementioned drugs, occasionally new drug candidates are identified that accomplish this seemingly difficult feat. Such is the case for a class of synthetic alkaloids whose birth and swift entrance in the medical field of anesthesiology originated with the synthesis of fentanyl (**4**, Fig. 1) by Paul Janssen in 1960 [8]–[11]. Since its synthesis, inspired partly by the necessity to improve the potency and bioavailability of the structurally related opiate Demerol (**3**), fentanyl analogs with superior pharmacokinetic properties, onset time, and effective dosage have been successfully produced [12], [13]. Currently, a significant array of fentanyl analogs exists spanning a large range of physicochemical properties, which strictly determine their ultimate application. Some of these compounds, along with their potency relative to morphine, are given in Fig. 2 [12], [14].

With drugs of this kind, propensity of their users to become physiologically dependent has been reported, and indeed there exist issues involving the use of fentanyl and its analogs [15], [16]. For example, these compounds have been the epicenter of fatal incidents involving overdoses by users who self-administer quantities that are just minimally beyond the carefully prescribed doses for controlling pain in a clinical setting. Additionally, there has been documented military misuse of these compounds for their crowd controlling properties. As a particularly infamous case, the presumed use of gaseous/aerosolized fentanyl derivatives by Russian security forces to incapacitate terrorists during a Moscow theater hostage crisis in 2002 led to the death of 170 people, 127 of them hostages [17]–[21]. The powerful effects of these compounds at such low doses combined with the lack of medical training in cases of illicit use make these drugs extremely dangerous outside the clinical environment.

Fentanyl (prescribed more commonly by its trade name Sublimaze) is approximately 50–100 times more potent than morphine, a quality that has righteously cemented this drug and its congeners in the medical field as the primary choice for a fast acting anesthetic during perioperative procedures. Their *modus operandi* is believed to involve the binding to the transmembrane µ-opioid receptors on cell surfaces resulting in a cascade of intracellular signals that eventually results in their biological effect [22], [23]. Even though to date a detailed description of this receptor binding event remains undiscovered, a suitable model can be proposed based on the known binding of similar opioids to various nociceptive/opioid receptors for which few crystallographic structures have been solved [24]–[27].

**Figure 3:**
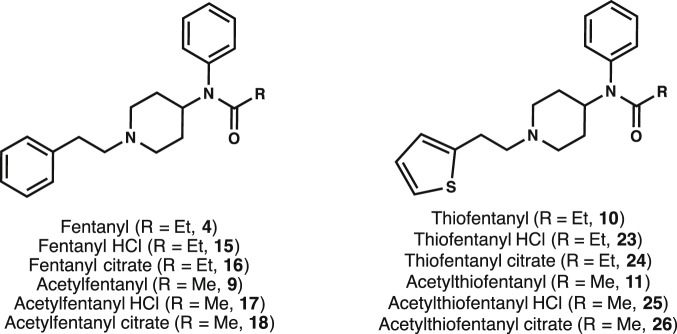
Fentanyl analogs synthesized in this work.

Due to the importance of this class of opioids in the biomedical field as well as their history of illicit use, it is not surprising that several synthetic routes have been devised for their construction since Janssen’s original disclosure [28]–[31]. However, most of these routes focus on specific transformations along the original sequence to eventually provide fentanyl (**4**) in moderate yields. The need to understand fentanyl receptor activation and to develop potential countermeasures for illicit use coupled to the lack of established procedures for procuring high quality materials in gram quantities prompted us to revisit and optimize the synthetic route for parent fentanyl **4** along with additional analogs. The routes described herein were optimized to obtain fentanyls in high yields using an efficient, three-step synthetic strategy. The four analogs that our efforts focused on are: fentanyl (**4**), acetylfentanyl (**9**), thiofentanyl (**10**) and acetylthiofentanyl (**11**). The opioids were synthesized as the free bases as well as their more clinically relevant hydrochloride and citric acid salts (Fig. 3).

## Materials and Methods

Solvents used during the syntheses were removed by using a Büchi rotary evaporator R-200 equipped with a Büchi heating bath B-490 and coupled to a KNF Laboport Neuberger UN820 vacuum pump. Analytical thin layer chromatography (TLC) was conducted on Agela Technologies silica gel glass plates coupled with detection ceric ammonium molybdate (CAM), exposure to iodine vapor and/or UV light (λ = 254 nm). ^1^H NMR (600 MHz) and ^13^C NMR (150 MHz) were recorded in CDCl_3_ and D_2_O. Spectra were obtained using a Bruker Avance III 600 MHz instrument equipped with a Bruker QNP 5 mm cryoprobe (Bruker Biospin, Billerica, MA) at 30.0±0.1°C. NMR data is reported as follows: chemical shift (δ) (parts per million, ppm); multiplicity: s (singlet), d (doublet), t (triplet), q (quartet) and br (broad); coupling constants (*J*) are given in Hertz (Hz). ^1^H NMR chemical shifts are calibrated with respect to residual chloroform in CDCl_3_ centered at 7.26 ppm, whereas for ^13^C NMR, the center peak for CDCl_3_, centered at 77.0 ppm, was used for the calibration. All NMR spectra can be found in Information S1. HRMS analyses were obtained at the Forensic Science Center at the Lawrence Livermore National Laboratory using either Chemical Ionization (CI) or Electrospray Ionization (ESI). Elemental analyses were conducted at Galbraith Laboratories (Knoxville, TN).

## Results and Discussion

**Figure 4:**
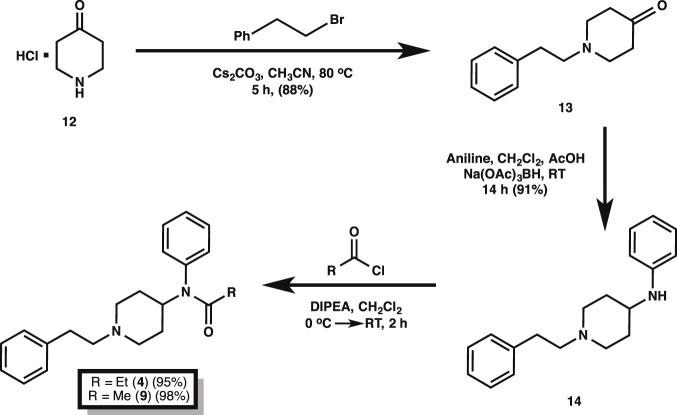
Synthesis of fentanyl and acetylthiofentanyl. Yields reflect the isolated materials by column chromatography after each step and using the optimized conditions (cf. Table 1). Citrate and hydrochloride salts for each analog were obtained in nearly quantitative yields by treating the free bases at the end of these routes with the corresponding acids.

**Figure 5:**
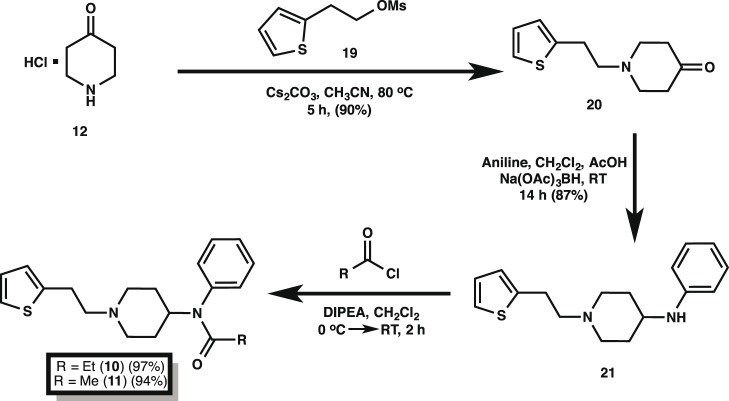
Synthesis of thiofentanyl and acetylthiofentanyl. Yields reflect the isolated materials by column chromatography after each step and using the optimized conditions (cf. Table 1). Citrate and hydrochloride salts for each analog were obtained in nearly quantitative yields by treating the free bases at the end of these routes with the corresponding acids.

Our final, optimized synthetic path to fentanyl (**4**) is outlined in Fig. 4 and it begins with the alkylation of commercially available 4-piperidone monohydrate hydrochloride **12** with 2-(bromoethyl)benzene in the presence of cesium carbonate to furnish alkylated piperidone **13** in 88% yield. Reductive amination with aniline of **13** mediated by sodium triacetoxyborohydride in the presence of acetic acid yielded the 4-piperidineamine precursor **14** in excellent yield (91%). Lastly, piperidineamine **14** was acylated using propionyl chloride in the presence of Hunig’s base to provide fentanyl (**4**) in 95% yield. Likewise, piperidineamine **14** was treated with acetic anhydride in the presence of Hunig’s base to provide acetylfentanyl (**9**) in 98% yield. Conversion of **4** and **9** into their hydrochloride and citrate salts proceeded smoothly in nearly quantitative yields (Fig. 3). The synthesis of the thiofentanyl analogs was accomplished in a similar fashion as shown in Fig. 5. Thus, 4-piperidone monohydrate hydrochloride **12** was alkylated with 2-(thiophen-2-yl)ethyl methanesulfonate (**19**) [32] in the presence of cesium carbonate to give *N*-[2-(2-thienyl)ethyl]-4-piperidinone (**20**) in 90% yield. Reductive amination with aniline of **20** with sodium triacetoxyborohydride and acetic acid yielded the 4-piperidineamine precursor **21** in 87% yield. Lastly, piperidineamine **21** was acylated using propionyl chloride to provide thiofentanyl (**10**) in 97% yield. Likewise, piperidineamine **21** was treated with acetic anhydride in the presence of Hunig’s base to provide acetylthiofentanyl (**11**) in 94% yield. As before, conversion of **10** and **11** to their respective hydrochloride and citric acid salts was accomplished smoothly in nearly quantitative yields (Fig. 3).

Due to the low-yielding characteristics of our initial attempts, we decided to explore optimization studies for the synthesis of fentanyl (**4**) and then apply these to the syntheses of the analogs. Several conditions for each one of the steps composing the overall sequence were considered and evaluated (Table 1). We deduced that optimal conditions discovered for the synthesis of **4** could be directly translated to the syntheses of fentanyls **9–11** as they all share a common synthetic pathway. Thus, it was found that the use of acetonitrile instead of dimethylformamide increased the yields of the first alkylation step from 72 to 88% (Table 1, entries 1 and 2). This was also observed during the synthesis of the thiofentanyl precursor (**20**) that made use of the mesylate (**19**) as the alkylating species where the yield markedly increased from 62 to 83% (Table 1, entries 3 and 4). For the reductive amination (RA) step, the need for an equimolar amount of acetic acid was noted as this resulted in the efficient conversion of ketone **13** into the piperidineamine precursor **14** in the presence of sodium triacetoxyborohydride (Table 1, entry 5) [33], [34]. Carrying out the reductive amination under the same conditions but switching the hydride source to either sodium cyanoborohydride or sodium borohydride resulted in significant loss of yield at room temperature (Table 1, entries 6 and 7). However, use of the latter hydride reagents under refluxing conditions (80°C) increased their yields significantly (Table 1, entries 8 and 9). Lastly, for the acylation step of the sequence, the use of either propanoyl chloride or propanoic anhydride resulted in nearly identical yields (95% *vs*. 94%) regardless of the solvent to carry out the transformation (pyridine or dichloromethane) (Table 1, entries 10–12).

**Table 1:** Optimization steps for the synthesis of fentanyl (**4**); *^a^*isolated yield; *^b^*alkylation in the synthesis of thiofentanyl derivatives; *^c^*reductive amination.

Entry	Synthetic Step	Reagents/Conditions	T (°C)	Yield*^a^* (%)
1	Alkylation	PhCH_2_CH_2_Br, Cs_2_CO_3_, DMF	80	72
2		PhCH_2_CH_2_Br, Cs_2_CO_3_, CH_3_CN	80	88
3		R-OMs (**19**), Cs_2_CO_3_, DMF*^b^*	80	62
4		R-OMs (**19**), Cs_2_CO_3_, CH_3_CN*^b^*	80	83
5	RA*^c^*	Na(OAc)_3_BH, CH_2_Cl_2_, AcOH	25	91
6		NaCNBH_3_, CH_2_Cl_2_, AcOH	25	64
7		NaBH_4_, CH_2_Cl_2_, AcOH	25	52
8		NaCNBH_3_, CH_2_Cl_2_, AcOH	80	86
9		NaBH_4_, CH_2_Cl_2_, AcOH	80	84
10	Acylation	Propanoyl anhydride, pyridine	25	94
11		Propanoyl chloride, pyridine	25	95
12		Propanoyl chloride, DIPEA, CH_2_Cl_2_	25	95

## Synthesis

### *N*-phenylethylpiperidin-4-one (13)

4-piperidone monohydrate hydrochloride (**12**) 22.0 g, 143.2 mmol) was dissolved in acetonitrile (400 mL) in a 1 L round-bottom flask equipped with a large stir bar and a condenser. The colorless solution was treated sequentially with cesium carbonate (Cs_2_CO_3_, 102.6 g, 315 mmol, 2.2 equiv.) and (2-bromoethyl)benzene (17.8 mL, 24.1 g, 130.2 mmol) at ambient temperature. The resulting suspension was vigorously stirred and refluxed at 80°C for 5 h. After 5 hours, the mixture was cooled to ambient temperature, transferred to a separatory funnel and partitioned (CH_2_Cl_2_//H_2_O). The organic phase was washed with brine (NaCl/H_2_O, 3×100 mL), satd. NaHCO_3_ (2×100 mL), dried over Na_2_SO_4_ and concentrated *in vacuo* to give a yellow oil. The oily mixture was purified by flash column chromatography (1∶1 → 7∶3 EtOAc/hexanes) to give **13** as a light yellow oil (23.3 g, 88%). R_f_ = 0.25 (1∶1 EtOAc/hexanes); ^1^H NMR (600 MHz, CDCl_3_) δ 7.31−7.28 (m, 2H), 7.22−7.19 (m, 3H), 2.85−2.83 (m, 2H), 2.82 (t, *J* = 6.0, 4H), 2.74−2.71 (m, 2H), 2.47 (t, *J* = 6.0, 4H); ^13^C NMR (150 MHz, CDCl_3_) δ 209.0, 140.0, 128.7, 128.4, 126.2, 59.3, 53.1, 41.2, 34.1; HRMS (CI) *m/z* calcd for C_13_H_17_NO [M^+^]: 203.1310; found 203.1309; Anal. Calcd for C_13_H_17_NO: C, 76.81; H, 8.43; N, 6.89. Found: C, 76.49; H, 8.71; N, 6.98.

### *N*-[1-(2-phenylethyl)-4-piperidinyl]aniline (14)

Aniline (8.1 mL, 8.24 g, 88.5 mmol) was taken up in methylene chloride (240 mL) in a 500 mL round-bottom flask equipped with a stir bar. The light brown solution was placed on an ice bath and treated dropwise with acetic acid (5.0 mL, 88.5 mmol). To the mixture, *N*-phenylethylpiperidin-4-one (**13**) (18.0 g, 88.5 mmol) was added as a solution in methylene chloride (60 mL), followed by the careful, slow addition of sodium triacetoxyborohydride (28.1 g, 132.8 mmol, 1.5 equiv.) in small portions. The reaction mixture was stirred at ambient temperature for 14 h. After this time, methanol (100 mL) was added to the mixture and all contents transferred to a separatory funnel. The mixture was partitioned (CH_2_Cl_2_//saturated NaHCO_3_). Once neutralized, the organic phase was washed with brine (NaCl/H_2_O, 3×100 mL), dried over Na_2_SO_4_ and concentrated *in vacuo* to give a light brown oil. The oily mixture was purified by flash column chromatography (1∶1 → 9∶1 EtOAc/hexanes) to give **14** as a light yellow oil (22.6 g, 91%). R_f_ = 0.22 (7∶3 EtOAc/hexanes); ^1^H NMR (600 MHz, CDCl_3_) δ 7.28−7.27 (m, 2H), 7.21−7.18 (m, 3H), 7.16−7.15 (m, 2H), 6.68 (tt, *J* = 7.2, 1.2, 1H), 6.61−6.59 (m, 2H), 3.50 (br, 1H), 3.33−3.30 (m, 1H), 2.96−2.94 (m, 2H), 2.83−2.80 (m, 2H), 2.63−2.60 (m, 2H), 2.21 (t, *J* = 12.0, 2H), 2.10−2.07 (m, 2H), 1.53−1.47 (m, 2H); ^13^C NMR (150 MHz, CDCl_3_) δ 147.1, 140.4, 129.3, 128.7, 128.4, 126.0, 117.2, 113.3, 60.7, 52.5, 50.2, 33.9, 32.6; HRMS (CI) *m/z* calcd for C_19_H_24_N_2_ [M^+^]: 280.1939; found 280.1937; Anal. Calcd for C_19_H_24_N_2_: C, 81.38; H, 8.63; N, 9.99. Found: C, 81.22; H, 8.68; N, 10.08.

### Fentanyl (4)

*N*-[1-(2-phenylethyl)-4-piperidinyl]aniline (**14**) (1.35 g, 4.8 mmol) was dissolved in methylene chloride (40 mL) in a 100 mL round bottom flask equipped with a small stir bar and was treated with diisopropylethylamine (DIPEA, 1.68 mL, 1.24 g, 9.6 mmol, 2.0 equiv.). The solution was cooled with an ice bath and treated dropwise with propionyl chloride (0.83 mL, 0.88 g, 9.6 mmol, 2.0 equiv.). The resulting mixture was stirred for 2 h at ambient temperature. The mixture was transferred to a separatory funnel and partitioned (CH_2_Cl_2_//H_2_O). The organic phase was washed with brine (NaCl/H_2_O, 1×50 mL), satd. NaHCO_3_ (1×50 mL), dried over anhydrous Na_2_SO_4_ and evaporated *in vacuo* at 40°C to give a yellow oil that was purified by flash column chromatography (3∶7 → 7∶3 EtOAc/hexanes) to furnish fentanyl (**4**) as a light yellow oil (1.53 g, 95%). R_f_ = 0.28 (1∶1 EtOAc/hexanes); ^1^H NMR (600 MHz, CDCl_3_) δ 7.48−7.37 (m, 3H), 7.33−7.27 (m, 2H), 7.25−7.17 (m, 3H), 7.13−7.05 (m, 2H), 4.88−4.71 (br, 1H), 3.83−3.47 (br, 2H), 3.20−3.09 (br, 2H), 3.09−2.99 (br, 2H), 2.82−2.70 (br, 2H), 2.13−1.99 (br, 4H), 1.94 (q, *J* = 7.4, 2H), 1.01 (t, *J* = 7.4, 3H); ^13^C NMR (150 MHz, CDCl_3_) δ 174.0, 138.1, 137.0, 129.9, 129.8, 129.0, 128.9, 128.7, 127.0, 59.1, 52.6, 50.7, 31.3, 28.4, 28.0, 9.5; HRMS (CI) *m/z* calcd for C_22_H_28_N_2_O [M^+^]: 336.2202; found 336.2201; Anal. Calcd for C_22_H_28_N_2_O: C, 78.53; H, 8.39; N, 8.33. Found: C, 78.42; H, 8.05; N, 8.62.

### Fentanyl hydrochloride (15)

Fentanyl (**4**) (138 mg, 0.41 mmol) was dissolved in diethyl ether (4 mL) in a 20 mL scintillation vial and treated at ambient temperature with 2.0 M HCl/Et_2_O solution (205 µL, 0.41 mmol) using a pipette. Upon addition of the acid, the colorless solution became a white suspension. The resulting suspension was stirred at ambient temperature for 2 hours and then filtered using suction filtration. The white solid was washed with diethyl ether (3×10 mL) and placed under vacuum overnight. Fentanyl hydrochloride (**15**) was obtained as a white solid (148 mg, 97%). ^1^H NMR (600 MHz, D_2_O) δ 7.53−7.50 (m, 3H), 7.36 (m, 2H), 7.32−7.24 (m, 5H), 4.77−4.61 (m, 1H) (overlaps with HOD), 3.66−3.61 (m, 2H), 3.32 (d, *J* = 8.4, 2H), 3.15 (d, *J* = 12.6, 2H), 3.00 (d, *J* = 8.4, 2H), 2.12−2.10 (m, 2H), 2.00 (q, *J* = 7.2, 2H), 1.64−1.58 (m, 2H), 0.92 (t, *J* = 7.2, 3H); ^13^C NMR (150 MHz, D_2_O) δ 177.5, 137.2, 136.3, 129.8, 129.3, 129.1, 128.8, 127.4, 57.6, 52.1, 50.0, 29.8, 28.2, 27.5, 9.0; TOF-MS (ESI) *m/z* calcd for C_22_H_29_N_2_O [M+H^+^]: 337.2274; found 337.2272; Anal. Calcd for C_22_H_29_ClN_2_O: C, 70.85; H, 7.84; N, 7.51. Found: C, 70.51; H, 8.03; N, 7.53.

### Fentanyl citrate (16)

Fentanyl (**4**) (148 mg, 0.44 mmol) was dissolved in MeOH (4 mL) in a 20 mL scintillation vial and treated with citric acid (85 mg, 0.44 mmol). The clear solution was stirred at ambient temperature for 2 hours. The methanol was removed *in vacuo* at 50°C to obtain a glassy solid that upon scrapping from the surface of the vial yielded a white solid that was placed under vacuum overnight. Fentanyl citrate (**16**) was obtained as a white solid (221 mg, 95%). ^1^H NMR (600 MHz, D_2_O) δ 7.51−7.46 (m, 3H), 7.35−7.32 (m, 2H), 7.28−7.27 (m, 1H), 7.25−7.23 (m, 2H), 7.22−7.20 (m, 1H), 4.77−4.61 (m, 1H) (overlaps with HOD), 3.60−3.57 (m, 2H), 3.30−3.27 (m, 2H), 3.11 (td, *J* = 12.6, 3.0, 2H), 2.98−2.96 (m, 2H), 2.94 (d, *J* = 15.6, 2H), 2.78 (d, *J* = 15.6, 2H), 2.09−2.08 (m, 2H), 1.97 (q, *J* = 7.2, 2H), 1.57 (qd, *J* = 13.2, 3.6, 2H), 0.89 (t, *J* = 7.2, 3H); ^13^C NMR (150 MHz, D_2_O) δ 177.5, 177.0, 173.6, 137.2, 136.3, 129.8, 129.3, 129.1, 128.8, 127.4, 73.4, 57.6, 52.1, 50.0, 43.3, 29.9, 28.2, 27.5, 9.0; HRMS (CI) *m/z* calcd for C_22_H_29_N_2_O [M+H^+^]: 337.2274; found 337.2284; Anal. Calcd for C_28_H_36_N_2_O_8_: C, 63.62; H, 6.86; N, 5.30. Found: C, 63.88; H, 7.02; N, 5.66.

### Acetylfentanyl (9)

*N*-[1-(2-phenylethyl)-4-piperidinyl]aniline (**14**) (1.50 g, 5.34 mmol) was dissolved in methylene chloride (50 mL) in a 100 mL round bottom flask with a small stir bar and was treated with diisopropylethylamine (DIPEA, 1.86 mL, 1.38 g, 10.7 mmol, 2.0 equiv.). The solution was cooled with an ice bath and treated dropwise with acetic anhydride (1.0 mL, 1.1 g, 10.7 mmol, 2.0 equiv.). The resulting mixture was stirred for 2 h at ambient temperature. The mixture was transferred to a separatory funnel and partitioned (CH_2_Cl_2_//H_2_O). The organic phase was washed with brine (NaCl/H_2_O, 1×50 mL), satd. NaHCO_3_ (1×50 mL), dried over anhydrous Na_2_SO_4_ and evaporated *in vacuo* at 40°C to give a yellow oil that was purified by flash column chromatography (3∶7 → 7∶3 EtOAc/hexanes) to furnish acetylfentanyl (**9**) as a light yellow oil (1.68 g, 98%). R_f_ = 0.28 (1∶1 EtOAc/hexanes); ^1^H NMR (600 MHz, CDCl_3_) δ 7.45−7.36 (m, 3H), 7.30−7.25 (m, 2H), 7.20−7.16 (m, 3H), 7.11−7.09 (m, 2H), 4.69 (tt, *J* = 12.0, 4.2, 1H), 3.42−3.36 (br, 1H), 3.04−3.00 (m, 2H), 2.80−2.73 (m, 2H), 2.60−2.55 (m, 2H), 2.20 (td, *J* = 12.0, 2.4, 2H), 1.86−1.82 (m, 2H), 1.77 (s, 3H), 1.47 (qd, *J* = 12.6, 4.2, 2H); ^13^C NMR (150 MHz, CDCl_3_) δ 170.3, 140.2, 139.3, 130.3, 129.4, 128.6, 128.4, 128.4, 126.1, 60.4, 53.0, 52.1, 33.7, 30.4, 23.5; HRMS (CI) *m/z* calcd for C_21_H_26_N_2_O [M^+^]: 322.2045; found 322.2043; Anal. Calcd for C_21_H_26_N_2_O: C, 78.22; H, 8.13; N, 8.69. Found: C, 78.26; H, 8.11; N, 8.75.

### Acetylfentanyl hydrochloride (17)

Acetylfentanyl (**9**) (156 mg, 0.48 mmol) was dissolved in diethyl ether (4 mL) in a 20 mL scintillation vial and treated at ambient temperature with 2.0 M HCl/Et_2_O solution (242 µL, 0.48 mmol) using a pipette. Upon addition of the acid, the colorless solution became a white suspension. The resulting suspension was stirred at ambient temperature for 2 hours and then filtered using suction filtration. The white solid was washed with diethyl ether (3×10 mL) and placed under vacuum overnight. Acetylfentanyl hydrochloride (**17**) was obtained as a white solid (165 mg, 96%). ^1^H NMR (600 MHz, D_2_O) δ 7.65−7.44 (m, 3H), 7.33−7.30 (m, 2H), 7.27−7.24 (m, 1H), 7.23−7.20 (m, 4H), 4.77−4.61 (m, 1H) (overlaps with HOD), 3.60−3.54 (m, 2H), 3.30−3.24 (m, 2H), 3.13−3.10 (m, 2H), 3.07−2.90 (m, 2H), 2.09−2.04 (m, 2H), 1.72 (s, 3H), 1.60−1.53 (m, 2H); ^13^C NMR (150 MHz, D_2_O) δ 174.1, 137.6, 136.2, 129.8, 129.7, 129.3, 129.1, 128.8, 127.4, 57.6, 52.1, 50.0, 29.9, 27.4, 22.5; TOF-MS (ESI) *m/z* calcd for C_21_H_27_N_2_O [M+H^+^]: 323.2118; found 323.2124; Anal. Calcd for C_21_H_27_ClN_2_O: C, 70.28; H, 7.58; N, 7.81. Found: C, 69.97; H, 7.72; N, 7.91.

### Acetylfentanyl citrate (18)

Acetylfentanyl (**9**) (150 mg, 0.46 mmol) was dissolved in MeOH (4 mL) in a 20 mL scintillation vial and treated with citric acid (90 mg, 0.46 mmol). The clear solution was stirred at ambient temperature for 2 hours. The methanol was removed *in vacuo* at 50°C to obtain a glassy solid that upon scrapping from the surface of the vial yielded a white solid that was placed under vacuum overnight. Fentanyl citrate (**18**) was obtained as a white solid (225 mg, 95%). ^1^H NMR (600 MHz, D_2_O) δ 7.53−7.47 (m, 3H), 7.36−7.33 (m, 2H), 7.30−7.26 (m, 1H), 7.26−7.22 (m, 4H), 4.77−4.61 (m, 1H) (overlaps with HOD), 3.62−3.58 (m, 2H), 3.31−3.28 (m, 2H), 3.11 (td, *J* = 13.2, 2.4, 2H), 2.99−2.96 (m, 2H), 2.87 (d, *J* = 15.6, 2H), 2.73 (d, *J* = 15.6, 2H), 2.12−2.10 (m, 2H), 1.74 (s, 3H), 1.58 (qd, *J* = 14.4, 3.0, 2H); ^13^C NMR (150 MHz, D_2_O) δ 178.3, 174.5, 174.1, 137.6, 136.3, 129.8, 129.7, 129.3, 129.1, 128.8, 127.4, 73.7, 57.6, 52.1, 49.9, 48.9, 43.6, 29.8, 27.4, 22.5; TOF-MS (ESI) *m/z* calcd for C_21_H_27_N_2_O [M+H^+^]: 323.2118; found 323.2127; Anal. Calcd for C_27_H_34_N_2_O_8_: C, 63.02; H, 6.66; N, 5.44. Found: C, 63.38; H, 6.69; N, 5.78.

### 2-(Thiophen-2-yl)ethyl methanesulfonate (19) [32]

2-Thiophene ethanol (5.0 g, 39 mmol) was taken up in methylene chloride (50 mL) in a 250 mL round bottom flask and treated with triethylamine (TEA, 6.5 mL, 46.8 mmol). The resulting dark brown solution was cooled with an ice bath and treated with mesyl chloride (MsCl, 3.7 mL, 46.8 mmol, 1.2 equiv.). The ice bath was removed and the resulting brown solution was allowed to warm to ambient temperature where it was stirred overnight. The brown solution was transferred to a separatory funnel and partitioned (CH_2_Cl_2_//H_2_O). The organic phase was washed with brine (NaCl/H_2_O, 3×50 mL), satd. NaHCO_3_ (1×50 mL), dried over anhydrous Na_2_SO_4_ and evaporated *in vacuo* at 50°C to give a brown oil that was purified by flash column chromatography (hexanes → 3∶7 EtOAc/hexanes) to furnish thiophene mesylate **19** as a light brown oil (6.76 g, 84%). R_f_ = 0.54 (3∶7 EtOAc/hexanes); ^1^H NMR (600 MHz, CDCl_3_) δ 7.91 (dd, *J* = 4.8, 0.6, 1H), 6.95 (dd, *J* = 5.4, 3.6, 1H), 6.91−6.90 (m, 1H), 4.42 (t, *J* = 6.6, 2H), 3.27 (t, *J* = 6.6, 2H), 2.92 (s, 3H); ^13^C NMR (150 MHz, CDCl_3_) δ 138.1, 127.1, 126.2, 124.5, 69.7, 37.4, 29.8; HRMS (CI) *m/z* calcd for C_7_H_10_O_3_S_2_ [M^+^]: 206.0071; found 206.0070.

### *N*-[2-(2-thienyl)ethyl]-4-piperidinone (20)

4-piperidone monohydrate hydrochloride (**12**) (2.22 g, 14.5 mmol) was dissolved in acetonitrile (80 mL) in a 250 mL round-bottom flask equipped with a stir bar and a condenser. The colorless solution was treated sequentially with cesium carbonate (Cs_2_CO_3_, 10.4 g, 31.9 mmol, 2.2 equiv.) and 2-(thiophen-2-yl)ethyl methanesulfonate (**19**) (3.0 g, 14.5 mmol) at ambient temperature. The resulting suspension was vigorously stirred and refluxed at 80°C for 5 h. After 5 hours, the mixture was cooled to ambient temperature, transferred to a separatory funnel and partitioned (CH_2_Cl_2_//H_2_O). The organic phase was washed with brine (NaCl/H_2_O, 3×50 mL), saturated NaHCO_3_ (2×50 mL), dried over Na_2_SO_4_ and concentrated *in vacuo* to give a yellow oil. The oily mixture was purified by flash column chromatography (1∶1 → 7∶3 EtOAc/hexanes) to give **20** as a light yellow oil (2.7 g, 90%). R_f_ = 0.25 (1∶1 EtOAc/hexanes); ^1^H NMR (600 MHz, CDCl_3_) δ 7.14 (dd, *J* = 5.4, 1.2, 1H), 6.92 (dd, *J* = 5.4, 3.6, 1H), 6.84 (dq, *J* = 3.6, 1.2, 1H), 3.04 (t, *J* = 7.2, 2H), 2.82 (t, *J* = 6.0, 4H), 2.77 (t, *J* = 7.2, 2H), 2.48 (t, *J* = 6.0, 4H); ^13^C NMR (150 MHz, CDCl_3_) δ 209.0, 142.4, 126.6, 124.7, 123.7, 60.4, 53.0, 41.3, 28.3; HRMS (CI) *m/z* calcd for C_11_H_15_NOS [M^+^]: 209.0874; found 209.0872; Anal. Calcd for C_11_H_15_NOS: C, 63.12; H, 7.22; N, 6.69; Found: C, 63.01; H, 7.16; N, 6.77.

### *N*-phenyl-1-(2-(thiophen-2-yl)ethyl)piperidin-4-amine (21)

Aniline (0.53 mL, 5.7 mmol) was taken up in methylene chloride (50 mL) in a 250 mL round-bottom flask equipped with a stir bar. The light brown solution was placed on an ice bath and treated dropwise with acetic acid (0.32 mL, 5.7 mmol). To the mixture, *N*-[2-(2-thienyl)ethyl]-4-piperidinone (**20**) (1.2 g, 5.7 mmol) was added as a solution in methylene chloride (30 mL), followed by the careful, slow addition of sodium triacetoxyborohydride (1.8 g, 8.6 mmol, 1.5 equiv.) in small portions. The reaction mixture was stirred at ambient temperature for 14 h. After this time, methanol (40 mL) was added to the mixture and all contents transferred to a separatory funnel. The mixture was partitioned (CH_2_Cl_2_//saturated NaHCO_3_). Once neutralized, the organic phase was washed with brine (NaCl/H_2_O, 3×50 mL), dried over Na_2_SO_4_ and concentrated *in vacuo* to give a light brown oil. The oily mixture was purified by flash column chromatography (1∶1 → 9∶1 EtOAc/hexanes) to give **21** as a light yellow oil (1.42 g, 87%). R_f_ = 0.33 (7∶3 EtOAc/hexanes); ^1^H NMR (600 MHz, CDCl_3_) δ 7.18−7.15 (m, 2H), 7.13 (dd, *J* = 5.4, 1.2, 1H), 6.92 (dd, *J* = 5.4, 3.6, 1H), 6.84−6.82 (m, 1H), 6.69 (tt, *J* = 7.2, 0.6, 1H), 6.62−6.58 (m, 1H), 3.52 (br, 1H), 3.34−3.31 (m, 1H), 2.13 (t, *J* = 6.6, 2H), 2.95−2.93 (m, 2H), 2.68 (t, *J* = 6.6, 2H), 2.22 (td, *J* = 13.2, 2.4, 2H), 2.10−2.07 (m, 2H), 1.54−1.51 (m, 2H); ^13^C NMR (150 MHz, CDCl_3_) δ 147.1, 142.9, 129.3, 126.6, 124.6, 123.5, 117.2, 113.3, 60.0, 52.4, 49.9, 32.6, 28.0; HRMS (CI) *m/z* calcd for C_17_H_22_N_2_S [M^+^]: 286.1504; found 286.1503; Anal. Calcd for C_17_H_22_N_2_S: C, 71.29; H, 7.74; N, 9.78; Found: C, 71.16; H, 7.75; N, 9.66.

### Thiofentanyl (10)

*N*-phenyl-1-(2-(thiophen-2-yl)ethyl)piperidin-4-amine (**21**) (2.21 g, 7.74 mmol) was dissolved in methylene chloride (50 mL) in a 100 mL round bottom flask equipped with a stir bar and was treated with diisopropylethylamine (DIPEA, 2.0 mL, 1.5 g, 11.6 mmol, 1.5 equiv.). The solution was cooled with an ice bath and treated dropwise with propionyl chloride (0.99 mL, 11.6 mmol). The resulting mixture was stirred for 2 h at ambient temperature. The mixture was transferred to a separatory funnel and partitioned (CH_2_Cl_2_//H_2_O). The organic phase was washed with brine (NaCl/H_2_O, 1×50 mL), satd. NaHCO_3_ (1×50 mL), dried over anhydrous Na_2_SO_4_ and evaporated *in vacuo* at 40°C to give a yellow oil that was purified by flash column chromatography (3∶7 → 7∶3 EtOAc/hexanes) to furnish thiofentanyl (**10**) as a light yellow oil (2.57 g, 97%). R_f_ = 0.28 (1∶1 EtOAc/hexanes); ^1^H NMR (600 MHz, CDCl_3_) δ 7.38−7.33 (m, 3H), 7.09−7.06 (m, 2H), 6.88 (dd, *J* = 4.8, 3.0, 1H), 6.77−6.75 (m, 1H), 4.67 (tt, *J* = 12.0, 4.2, 1H), 2.97−2.92 (m, 4H), 2.61−2.58 (m, 2H), 2.17 (td, *J* = 12.0, 1.8, 2H), 1.91 (q, *J* = 7.8, 2H), 1.81−1.78 (br s, 1H), 1.40 (qd, *J* = 11.4, 3.6, 2H), 1.00 (t, *J* = 7.8, 3H); ^13^C NMR (150 MHz, CDCl_3_) δ 173.5, 142.6, 138.8, 130.4, 129.3, 128.3, 126.6, 124.5, 123.4, 60.0, 53.0, 52.1, 30.5, 28.5, 27.8, 9.6; HRMS (CI) *m/z* calcd for C_20_H_26_N_2_OS [M^+^]: 342.1766; found 342.1765; Anal. Calcd for C_20_H_26_N_2_OS: C, 70.14; H, 7.65; N, 8.18; Found: C, 70.11; H, 7.63; N, 8.10.

### Thiofentanyl hydrochloride (23)

Thiofentanyl (**10**) (300 mg, 0.87 mmol) was dissolved in diethyl ether (6 mL) in a 20 mL scintillation vial and treated at ambient temperature with 2.0 M HCl/Et_2_O solution (435 µL, 0.87 mmol) using a pipette. Upon addition of the acid, the colorless solution became a white suspension. The resulting suspension was stirred at ambient temperature for 2 hours and then filtered using suction filtration. The white solid was washed with diethyl ether (3×10 mL) and placed under vacuum overnight. Thiofentanyl hydrochloride (**23**) was obtained as a white solid (319 mg, 97%). ^1^H NMR (600 MHz, D_2_O) δ 7.52−7.47 (m, 3H), 7.30 (dd, *J* = 5.4, 1.2, 1H), 7.25−7.22 (m, 2H), 6.98 (dd, *J* = 4.8, 3.0, 1H), 6.94−6.93 (m, 1H), 3.59−3.57 (m, 2H), 3.35 (t, *J* = 7.2, 2H), 3.23 (t, *J* = 7.2, 2H), 3.17−3.15 (m, 2H), 2.09−2.08 (m, 2H), 1.98 (q, *J* = 7.8, 2H), 1.62−1.58 (m, 2H), 0.90 (t, *J* = 7.8, 3H); ^13^C NMR (150 MHz, D_2_O) δ 177.5, 138.1, 137.2, 129.8, 129.3, 127.6, 126.5, 125.3, 57.6, 50.0, 28.2, 27.4, 24.2, 9.0; TOF-MS (ESI) *m/z* calcd for C_20_H_26_N_2_OS [M+H^+^]: 343.1839; found 343.1812; Anal. Calcd for C_20_H_27_ClN_2_OS: C, 63.39; H, 7.18; N, 7.39; Found: C, 63.14; H, 7.25; N, 7.42.

### Thiofentanyl citrate (24)

Thiofentanyl (**10**) (360 mg, 1.05 mmol) was dissolved in MeOH (5 mL) in a 20 mL scintillation vial and treated with citric acid (201 mg, 1.05 mmol). The clear solution was stirred at ambient temperature for 2 hours. The methanol was removed *in vacuo* at 50°C to obtain a glassy solid that upon scrapping from the surface of the vial yielded a white solid that was placed under vacuum overnight. Thiofentanyl citrate (**24**) was obtained as a white solid (538 mg, 96%). ^1^H NMR (600 MHz, D_2_O) δ 7.51−7.49 (m, 3H), 7.30 (dd, *J* = 4.8, 1.2, 1H), 7.23 (m, 2H), 6.98 (dd, *J* = 4.8, 3.6, 1H), 6.94−6.93 (m, 1H), 3.60−3.58 (m, 2H), 3.35 (t, *J* = 7.8, 2H), 3.24 (t, *J* = 7.8, 2H), 3.15−3.11 (m, 2H), 2.84 (d, *J* = 15.6, 2H), 2.72 (d, *J* = 15.6, 2H), 2.10−2.08 (m, 2H), 1.98 (q, *J* = 7.2, 2H), 1.59 (qd, *J* = 13.8, 3.6, 2H), 0.90 (t, *J* = 7.2, 3H); ^13^C NMR (150 MHz, D_2_O) δ 178.6, 177.5, 174.7, 138.1, 137.2, 129.8, 129.8, 129.3, 127.6, 126.5, 125.3, 73.8, 57.6, 52.1, 49.9, 28.2, 27.4, 24.1, 9.0; TOF-MS (ESI) *m/z* calcd for C_20_H_26_N_2_OS [M+H^+^]: 343.1839; found 343.1842; Anal. Calcd for C_20_H_27_ClN_2_OS: C, 58.41; H, 6.41; N, 5.24; Found: C, 58.70; H, 6.55; N, 5.41.

### Acetylthiofentanyl (11)

*N*-phenyl-1-(2-(thiophen-2-yl)ethyl)piperidin-4-amine (**21**) (1.2 g, 4.2 mmol) was dissolved in methylene chloride (50 mL) in a 100 mL round bottom flask equipped with a stir bar and was treated with diisopropylethylamine (DIPEA, 0.98 mL, 0.72 g, 8.4 mmol). The solution was cooled with an ice bath and treated dropwise with acetic anhydride (0.52 mL, 0.56 g, 8.4 mmol). The resulting mixture was stirred for 2 h at ambient temperature. The mixture was transferred to a separatory funnel and partitioned (CH_2_Cl_2_//H_2_O). The organic phase was washed with brine (NaCl/H_2_O, 1×10 mL), satd. NaHCO_3_ (1×10 mL), dried over anhydrous Na_2_SO_4_ and evaporated *in vacuo* at 40°C to give a yellow oil that was purified by flash column chromatography (3∶7 → 7∶3 EtOAc/hexanes) to furnish acetylthiofentanyl (**11**) as a light yellow oil (1.3 g, 94%). R_f_ = 0.28 (1∶1 EtOAc/hexanes); ^1^H NMR (600 MHz, CDCl_3_) δ 7.40−7.34 (m, 3H), 7.09−7.07 (m, 3H), 6.88 (d, *J* = 5.4, 3.6, 1H), 6.77−6.75 (m, 1H), 4.66 (tt, *J* = 12.0, 4.2, 1H), 2.99−2.93 (m, 4H), 2.62−2.58 (m, 2H), 2.19 (td, *J* = 12.6, 2.4, 2H), 1.81−1.79 (m, 2H), 1.74 (s, 3H), 1.43 (qd, *J* = 12.0, 3.6, 2H); ^13^C NMR (150 MHz, CDCl_3_) δ 170.3, 142.5, 139.4, 130.2, 129.4, 128.4, 126.7, 124.6, 123.4, 59.9, 52.9, 52.1, 30.4, 27.7, 23.5; HRMS (CI) *m/z* calcd for C_19_H_24_N_2_OS [M^+^]: 328.1609; found 328.1613; Anal. Calcd for C_19_H_24_N_2_OS: C, 69.48; H, 7.36; N, 8.53; Found: C, 69.44; H, 7.28; N, 8.46.

### Acetylthiofentanyl hydrochloride (25)

Acetylthiofentanyl (**11**) (260 mg, 0.79 mmol) was dissolved in diethyl ether (3 mL) in a 20 mL scintillation vial and treated at ambient temperature with 2.0 M HCl/Et_2_O solution (396 µL, 0.79 mmol) using a pipette. Upon addition of the acid, the colorless solution became a white suspension. The resulting suspension was stirred at ambient temperature for 2 hours and then filtered using suction filtration. The white solid was washed with diethyl ether (2×5 mL) and placed under vacuum overnight. Acetylthiofentanyl hydrochloride (**25**) was obtained as a white solid (273 mg, 95%). ^1^H NMR (600 MHz, D_2_O) δ 7.50−7.45 (m, 3H), 7.27 (dd, *J* = 5.4, 2.1, 1H), 7.22−7.21 (m, 2H), 6.95 (dd, *J* = 4.8, 3.6, 1H), 6.92−6.90 (m, 1H), 3.57−3.56 (m, 2H), 3.33 (t, *J* = 7.2, 2H), 3.21 (t, *J* = 7.2, 2H), 3.13−3.10 (m, 2H), 2.08−2.06 (m, 2H), 1.72 (s, 3H), 1.61−1.56 (m, 2H); ^13^C NMR (150 MHz, D_2_O) δ 174.1, 138.0, 137.6, 129.8, 129.6, 129.3, 127.5, 126.4, 125.3, 57.5, 52.1, 49.9, 27.3, 24.1, 22.4; TOF-MS (ESI) *m/z* calcd for C_19_H_25_N_2_OS [M+H^+^]: 329.1682; found 329.1666; Anal. Calcd for C_19_H_25_ClN_2_OS: C, 62.53; H, 6.91; N, 7.68; Found: C, 62.25; H, 7.00; N, 7.63.

### Acetylthiofentanyl citrate (26)

Acetylthiofentanyl (**11**) (282 mg, 0.86 mmol) was dissolved in MeOH (3 mL) in a 20 mL scintillation vial and treated with citric acid (165 mg, 0.86 mmol). The clear solution was stirred at ambient temperature for 2 hours. The methanol was removed *in vacuo* at 50°C to obtain a white solid that was placed under vacuum overnight. Acetylthiofentanyl citrate (**26**) was obtained as a white solid (438 mg, 98%). ^1^H NMR (600 MHz, D_2_O) δ 7.49−7.44 (m, 3H), 7.26 (dd, *J* = 4.8, 1.2, 1H), 7.20−7.19 (m, 2H), 6.94 (dd, *J* = 4.8, 3.0, 1H), 6.91−6.90 (m, 1H), 3.56−3.54 (m, 2H), 3.31 (t, *J* = 7.8, 2H), 3.20 (t, *J* = 7.2, 2H), 3.11−3.07 (m, 2H), 2.80 (d, *J* = 15.6, 2H), 2.68 (d, *J* = 15.6, 2H), 2.07−2.04 (m, 2H), 1.70 (s, 3H), 1.58−1.52 (m, 2H); ^13^C NMR (150 MHz, D_2_O) δ 178.3, 174.4, 174.1, 138.1, 137.5, 129.8, 129.6, 129.3, 127.5, 126.4, 125.2, 73.7, 57.5, 52.0, 49.3, 43.5, 27.3, 24.1, 22.4; TOF-MS (ESI) *m/z* calcd for C_19_H_25_N_2_OS [M+H^+^]: 329.1682; found 329.1688; Anal. Calcd for C_25_H_32_N_2_O_8_S: C, 57.68; H, 6.20; N, 5.38; Found: C, 57.83; H, 6.52; N, 5.53.

## Conclusion

The efficient syntheses of fentanyl and three other analogs (along with their hydrochloride and citric acid salts) have been accomplished. The three-step synthetic route was subject to optimization studies furnishing a process that generates the target fentanyls in high yields (73−78%). Thus, the syntheses described herein provide an efficient protocol for the construction of these interesting opioids for in depth biochemical as well as crystallographic studies.

## Supporting Information

Information S1 **Proton (^1^H) and Carbon (^13^C) NMR spectra for the fentanyl panel (free bases and salts) and their synthetic intermediates.** A more specific table of contents can be located in the document.
(PDF)
