# Supplementary material for: An Efficient, Optimized Synthesis of Fentanyl and Related Analogs
Source: PLoS One. 2014 Sep 18;9(9):e108250. doi: 10.1371/journal.pone.0108250 (PMC4169472; doi:10.1371/journal.pone.0108250)
Supplement: Information S1 — Proton (1H) and Carbon (13C) NMR spectra for the fentanyl panel (free bases and salts) and their synthetic intermediates. A more specific table of contents can be located in the document. (PDF) [file pone.0108250.s001.pdf]

An efficient, optimized synthesis of fentanyl and related analogs

Carlos A. Valdez, Roald N. Leif and Brian P. Mayer*

Forensic Science Center, Lawrence Livermore National Laboratory
Livermore, CA 94550 USA

- Supporting Information -

**Content**	**Page**
^{1}H NMR of N-phenylethylpiperidin-4-one (**13**)	S2
^{13}C NMR of N-phenylethylpiperidin-4-one (**13**)	S3
^{1}H NMR of N-[1-(2-phenylethyl)-4-piperidinyl]aniline (**14**)	S4
^{13}C NMR of N-[1-(2-phenylethyl)-4-piperidinyl]aniline (**14**)	S5
^{1}H NMR of Fentanyl (**4**)	S6
^{13}C NMR of Fentanyl (**4**)	S7
^{1}H NMR of Fentanyl hydrochloride (**15**)	S8
^{13}C NMR of Fentanyl hydrochloride (**15**)	S9
^{1}H NMR of Fentanyl citrate (**16**)	S10
^{13}C NMR of Fentanyl citrate (**16**)	S11
^{1}H NMR of Acetylfentanyl (**9**)	S12
^{13}C NMR of Acetylfentanyl (**9**)	S13
^{1}H NMR of Acetylfentanyl hydrochloride (**17**)	S14
^{13}C NMR of Acetylfentanyl hydrochloride (**17**)	S15
^{1}H NMR of Acetylfentanyl citrate (**18**)	S16
^{13}C NMR of Acetylfentanyl citrate (**18**)	S17
^{1}H NMR of 2-(Thiophen-2-yl)ethyl methanesulfonate (**19**)	S18
^{13}C NMR of 2-(Thiophen-2-yl)ethyl methanesulfonate (**19**)	S19
^{1}H NMR of N-[2-(2-thienyl)ethyl]-4-piperidinone (**20**)	S20
^{13}C NMR of N-[2-(2-thienyl)ethyl]-4-piperidinone (**20**)	S21
^{1}H NMR of N-phenyl-1-(2-(thiophen-2-yl)ethyl)piperidin-4-amine (**21**)	S22
^{13}C NMR of N-phenyl-1-(2-(thiophen-2-yl)ethyl)piperidin-4-amine (**21**)	S23
^{1}H NMR of Thiofentanyl (**10**)	S24
^{13}C NMR of Thiofentanyl (**10**)	S25
^{1}H NMR of Thiofentanyl hydrochloride (**23**)	S26
^{13}C NMR of Thiofentanyl hydrochloride (**23**)	S27
^{1}H NMR of Thiofentanyl citrate (**24**)	S28
^{13}C NMR of Thiofentanyl citrate (**24**)	S29
^{1}H NMR of Acetylthiofentanyl (**11**)	S30
^{13}C NMR of Acetylthiofentanyl (**11**)	S31
^{1}H NMR of Acetylthiofentanyl hydrochloride (**25**)	S32
^{13}C NMR of Acetylthiofentanyl hydrochloride (**25**)	S33
^{1}H NMR of Acetylthiofentanyl citrate (**26**)	S34
^{13}C NMR of Acetylthiofentanyl citrate (**26**)	S35

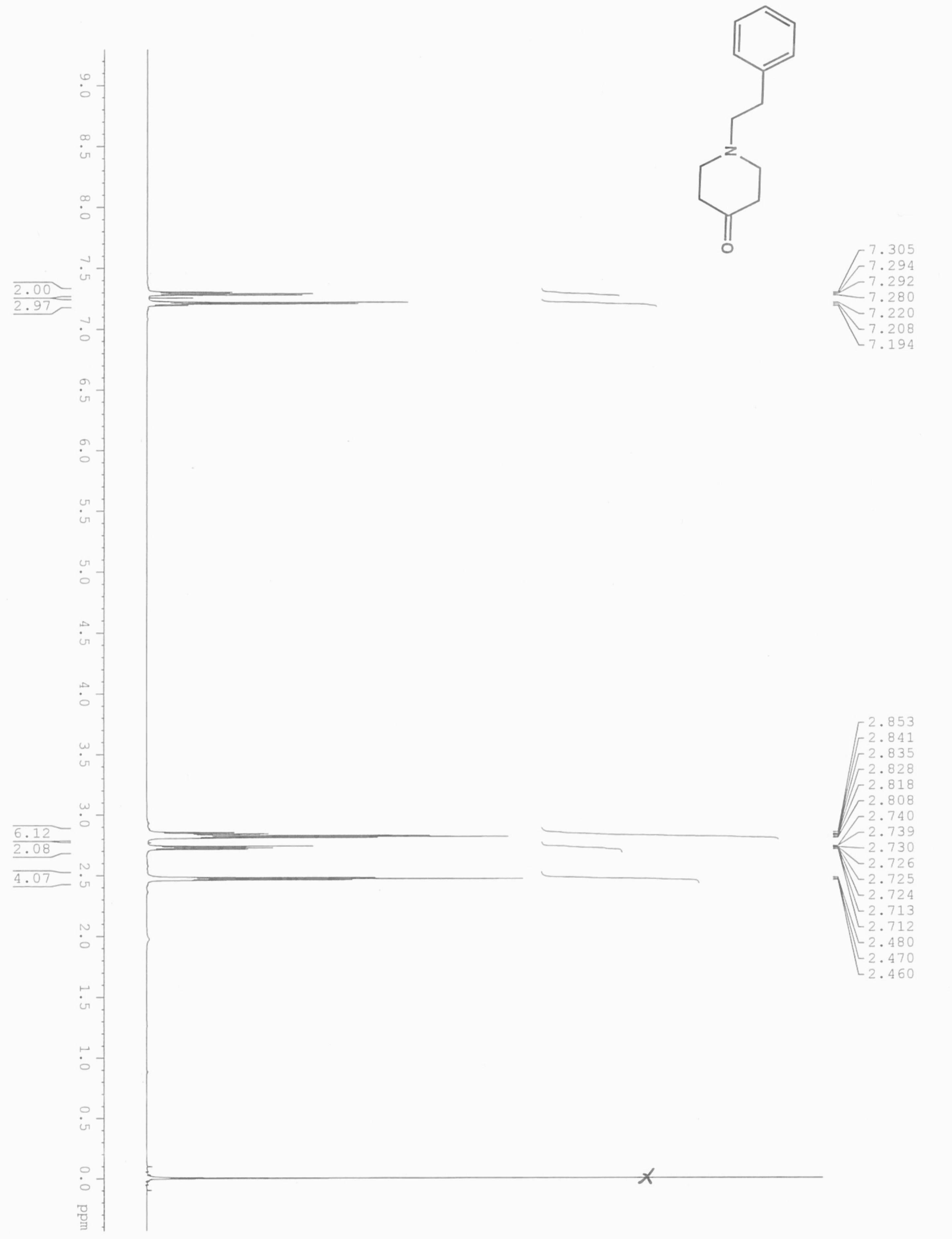
7.305
7.294
7.292
7.280
7.220
7.208
7.194
2.853
2.841
2.835
2.828
2.818
2.808
2.740
2.739
2.730
2.726
2.725
2.724
2.713
2.712
2.480
2.470
2.460
2.00
2.97
6.12
2.08
4.07
9.0
8.5
8.0
7.5
7.0
6.5
6.0
5.5
5.0
4.5
4.0
3.5
3.0
2.5
2.0
1.5
1.0
0.5
0.0
ppm

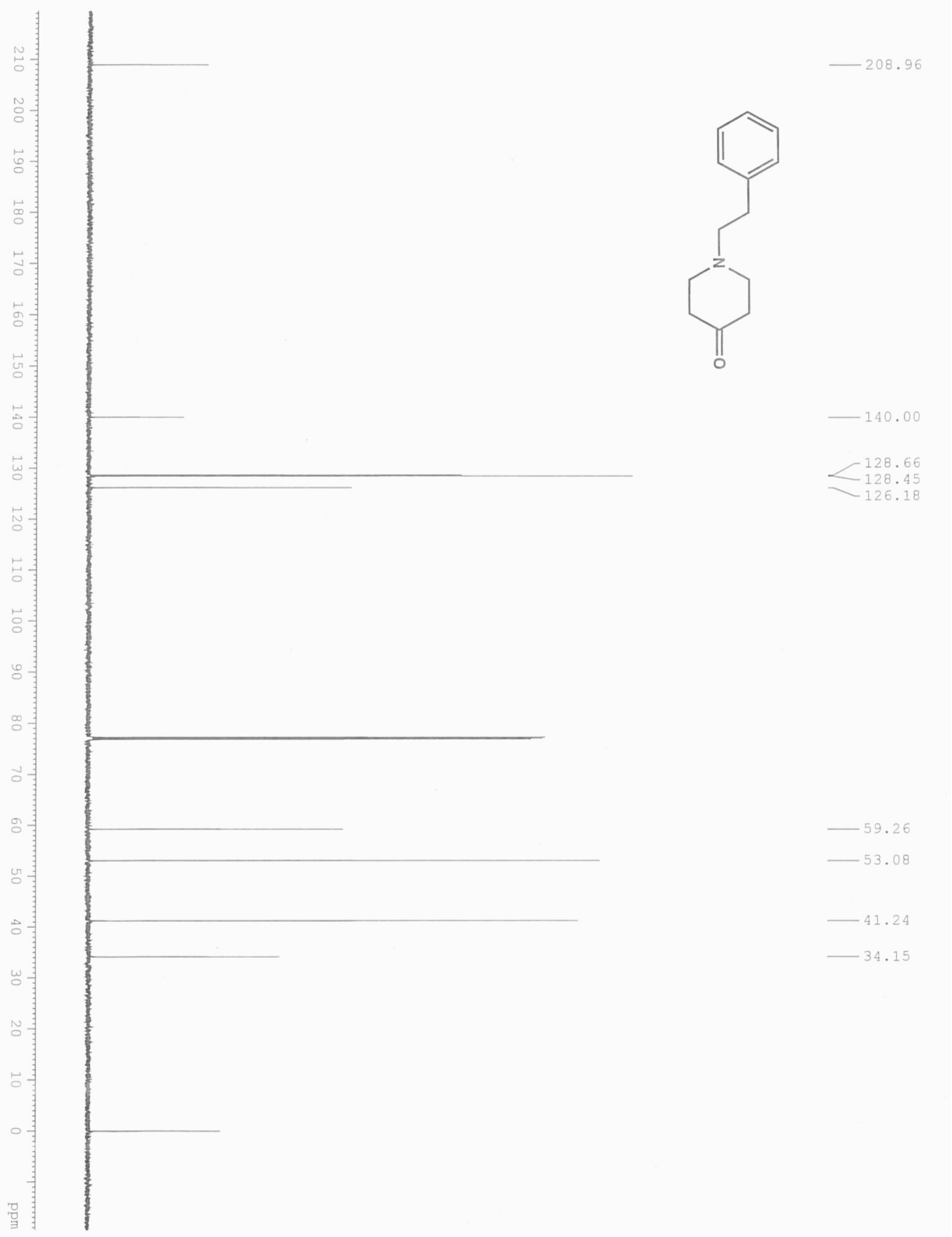
208.96
140.00
128.66
128.45
126.18
59.26
53.08
41.24
34.15
O
N
ppm

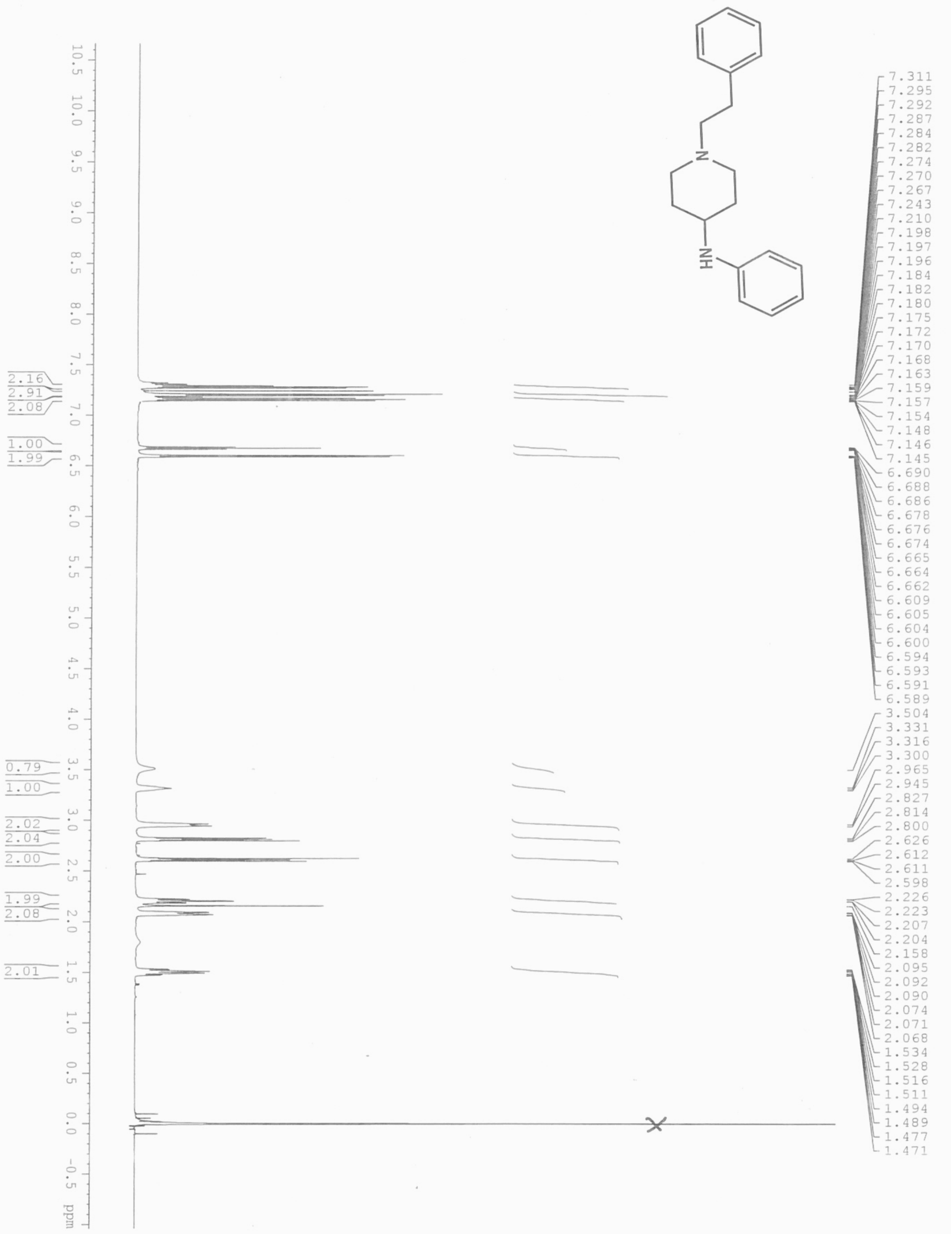
7.311
7.295
7.292
7.287
7.284
7.282
7.274
7.270
7.267
7.243
7.210
7.198
7.197
7.196
7.184
7.182
7.180
7.175
7.172
7.170
7.168
7.163
7.159
7.157
7.154
7.148
7.146
7.145
6.690
6.688
6.686
6.678
6.676
6.674
6.665
6.664
6.662
6.609
6.605
6.604
6.600
6.594
6.593
6.591
6.589
3.504
3.331
3.316
3.300
2.965
2.945
2.827
2.814
2.800
2.626
2.612
2.611
2.598
2.226
2.223
2.207
2.204
2.158
2.095
2.092
2.090
2.074
2.071
2.068
1.534
1.528
1.516
1.511
1.494
1.489
1.477
1.471
NH
N
2.16
2.91
2.08
1.00
1.99
0.79
1.00
2.02
2.04
2.00
1.99
2.08
2.01
ppm

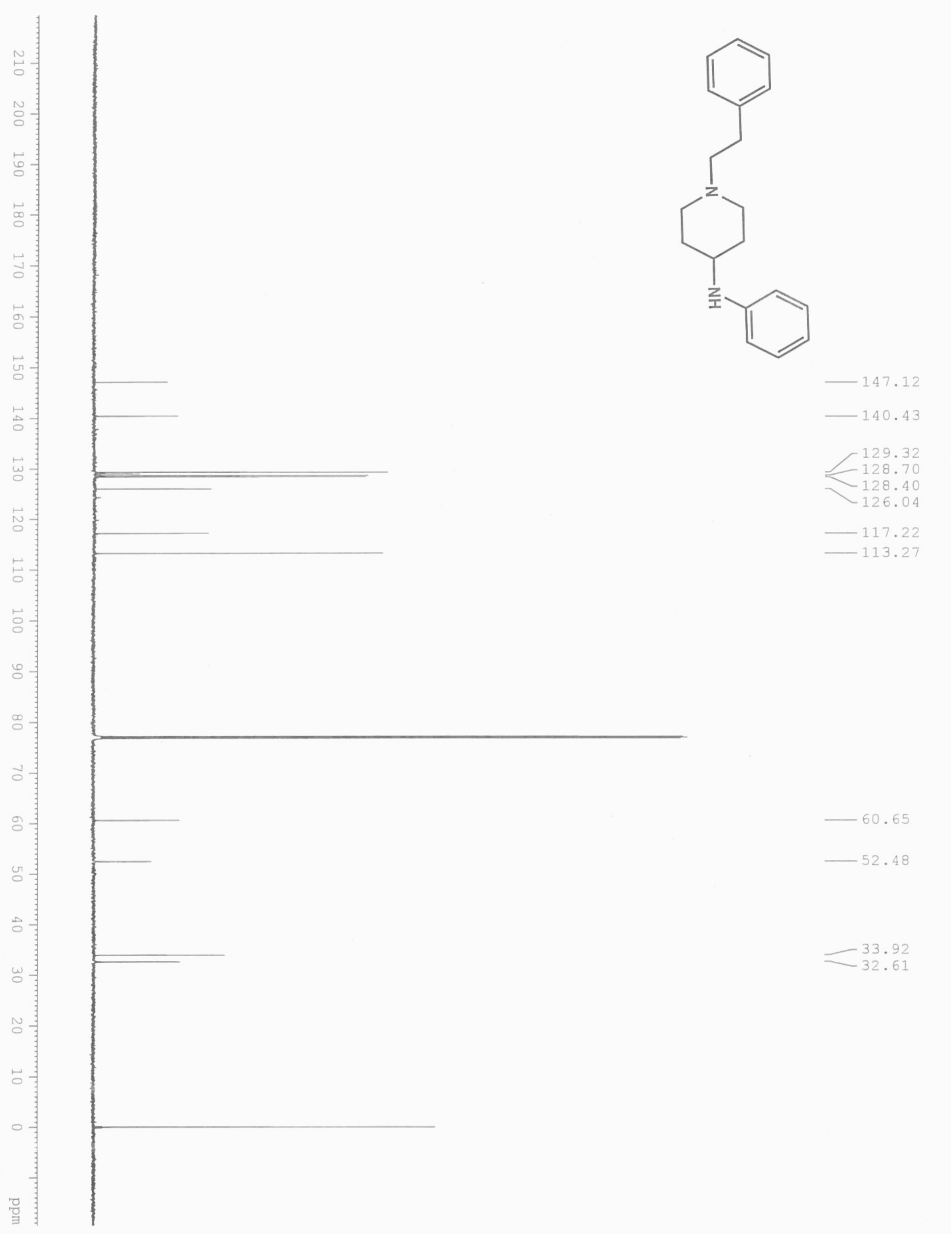

N
NH
147.12
140.43
129.32
128.70
128.40
126.04
117.22
113.27
60.65
52.48
33.92
32.61
ppm

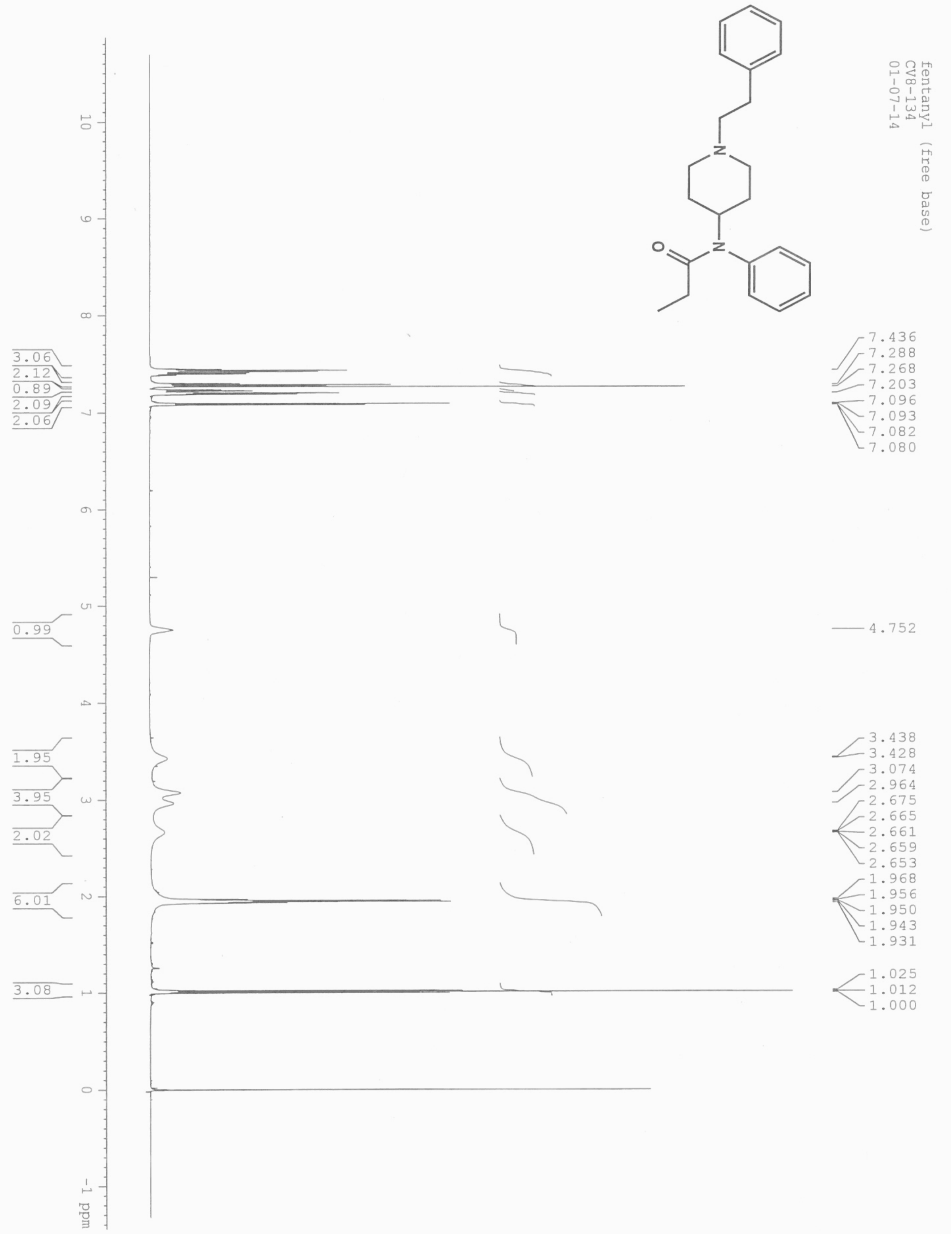
fentanyl (free base)
CV8-134
01-07-14
7.436
7.288
7.268
7.203
7.096
7.093
7.082
7.080
4.752
3.438
3.428
3.074
2.964
2.675
2.665
2.661
2.659
2.653
1.968
1.956
1.950
1.943
1.931
1.025
1.012
1.000
3.06
2.12
0.89
2.09
2.06
0.99
1.95
3.95
2.02
6.01
3.08
10
9
8
7
6
5
4
3
2
1
0
-1 ppm

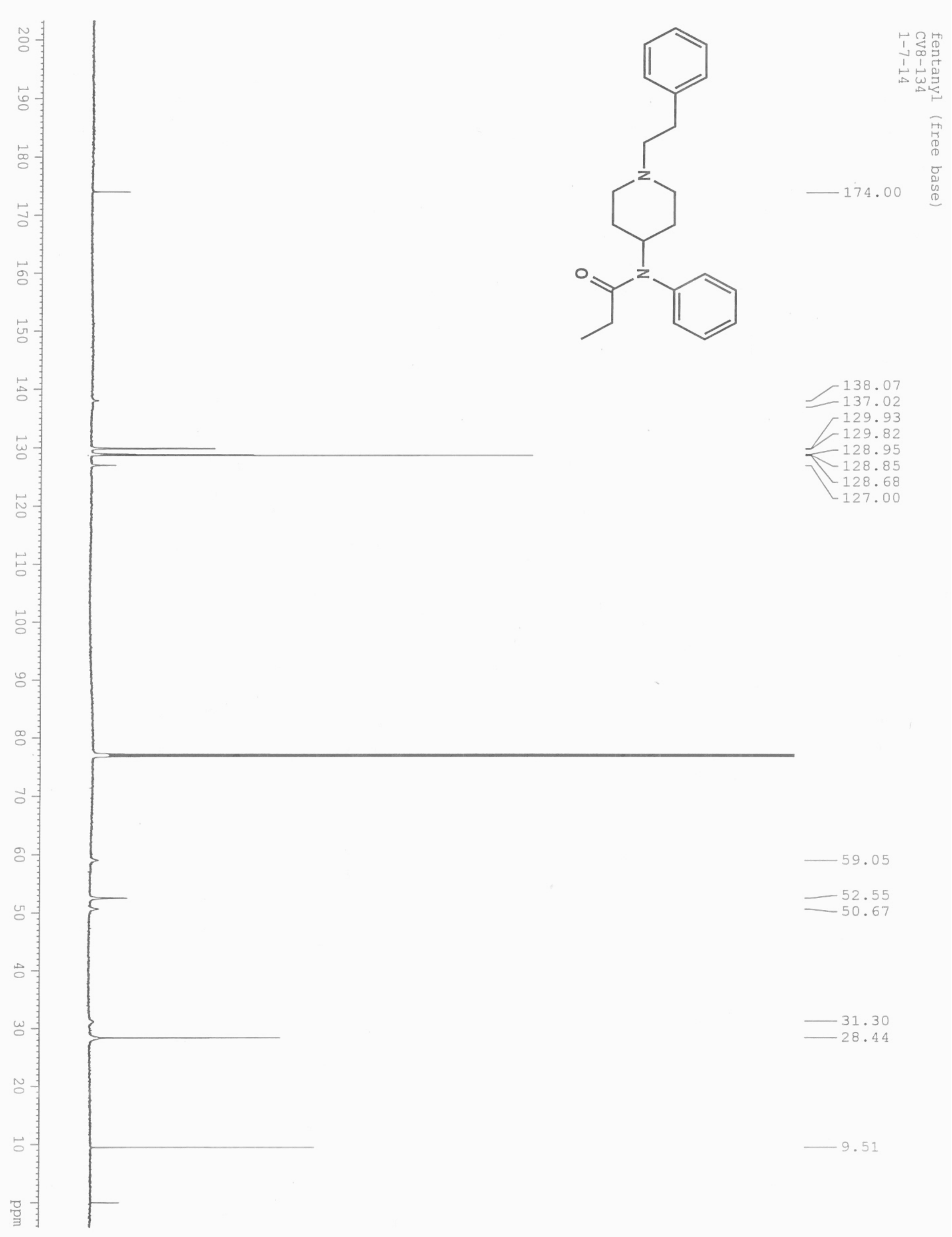
fentanyl (free base)
CV8-134
1-7-14
174.00
138.07
137.02
129.93
129.82
128.95
128.85
128.68
127.00
59.05
52.55
50.67
31.30
28.44
9.51
200 190 180 170 160 150 140 130 120 110 100 90 80 70 60 50 40 30 20 10
ppm

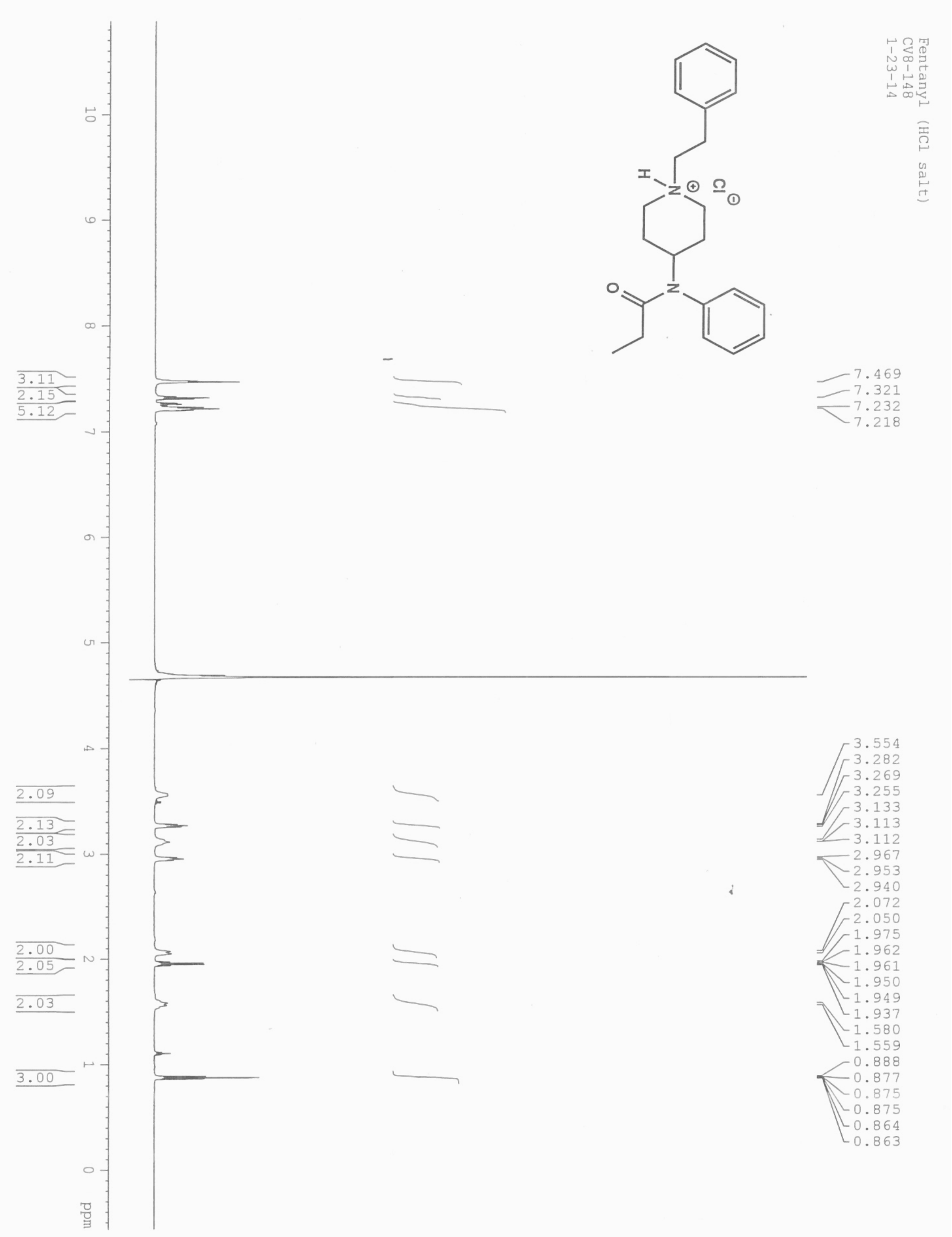

Fentanyl (HCl salt)
CV8-148
1-23-14
7.469
7.321
7.232
7.218
3.554
3.282
3.269
3.255
3.133
3.113
3.112
2.967
2.953
2.940
2.072
2.050
1.975
1.962
1.961
1.950
1.949
1.937
1.580
1.559
0.888
0.877
0.875
0.875
0.864
0.863
3.11
2.15
5.12
2.09
2.13
2.03
2.11
2.00
2.05
2.03
3.00
ppm

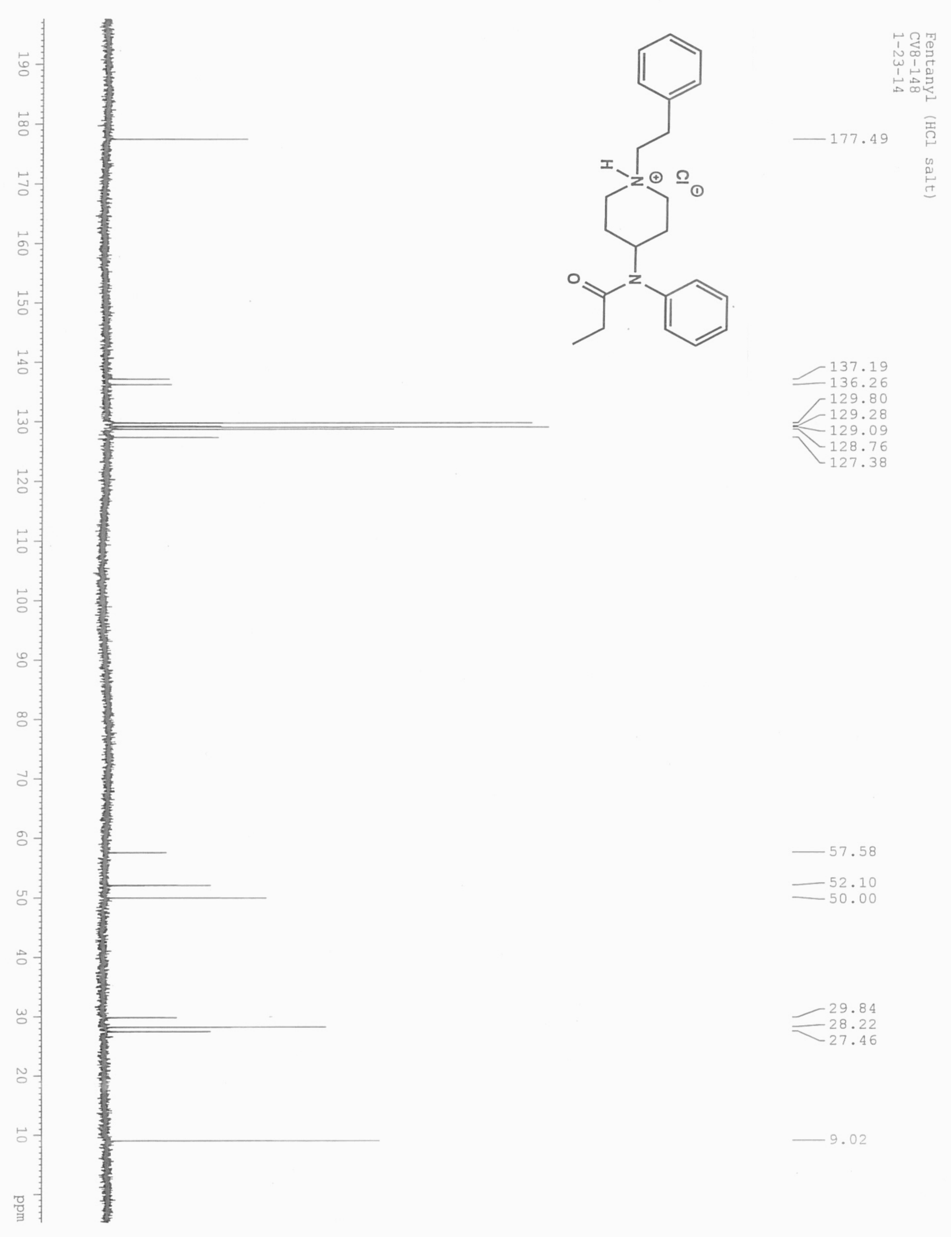
Fentanyl (HCl salt)
CV8-148
1-23-14
177.49
137.19
136.26
129.80
129.28
129.09
128.76
127.38
57.58
52.10
50.00
29.84
28.22
27.46
9.02
190
180
170
160
150
140
130
120
110
100
90
80
70
60
50
40
30
20
10
ppm

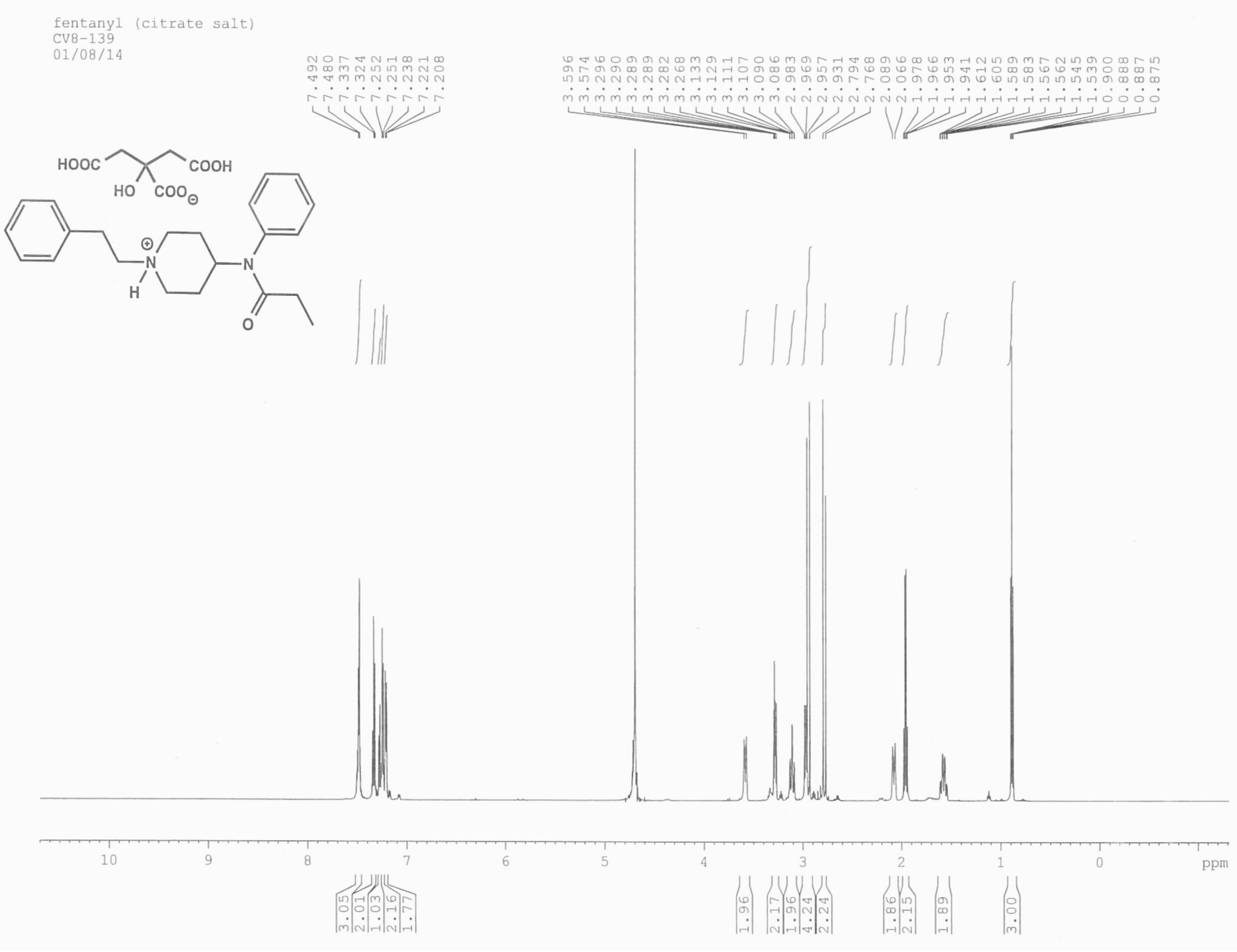
fentanyl (citrate salt)
CV8-139
01/08/14
HOOC
COOH
HO
COO
N
H
N
O
7.492
7.480
7.337
7.324
7.252
7.251
7.238
7.221
7.208
3.596
3.574
3.296
3.290
3.289
3.289
3.282
3.268
3.133
3.129
3.111
3.107
3.090
3.086
2.983
2.969
2.957
2.931
2.794
2.768
2.089
2.066
1.978
1.966
1.953
1.941
1.612
1.605
1.589
1.583
1.567
1.562
1.545
1.539
0.900
0.888
0.887
0.875
10
9
8
7
6
5
4
3
2
1
0
ppm
3.05
2.01
1.03
2.16
1.77
1.96
2.17
1.96
4.24
2.24
1.86
2.15
1.89
3.00

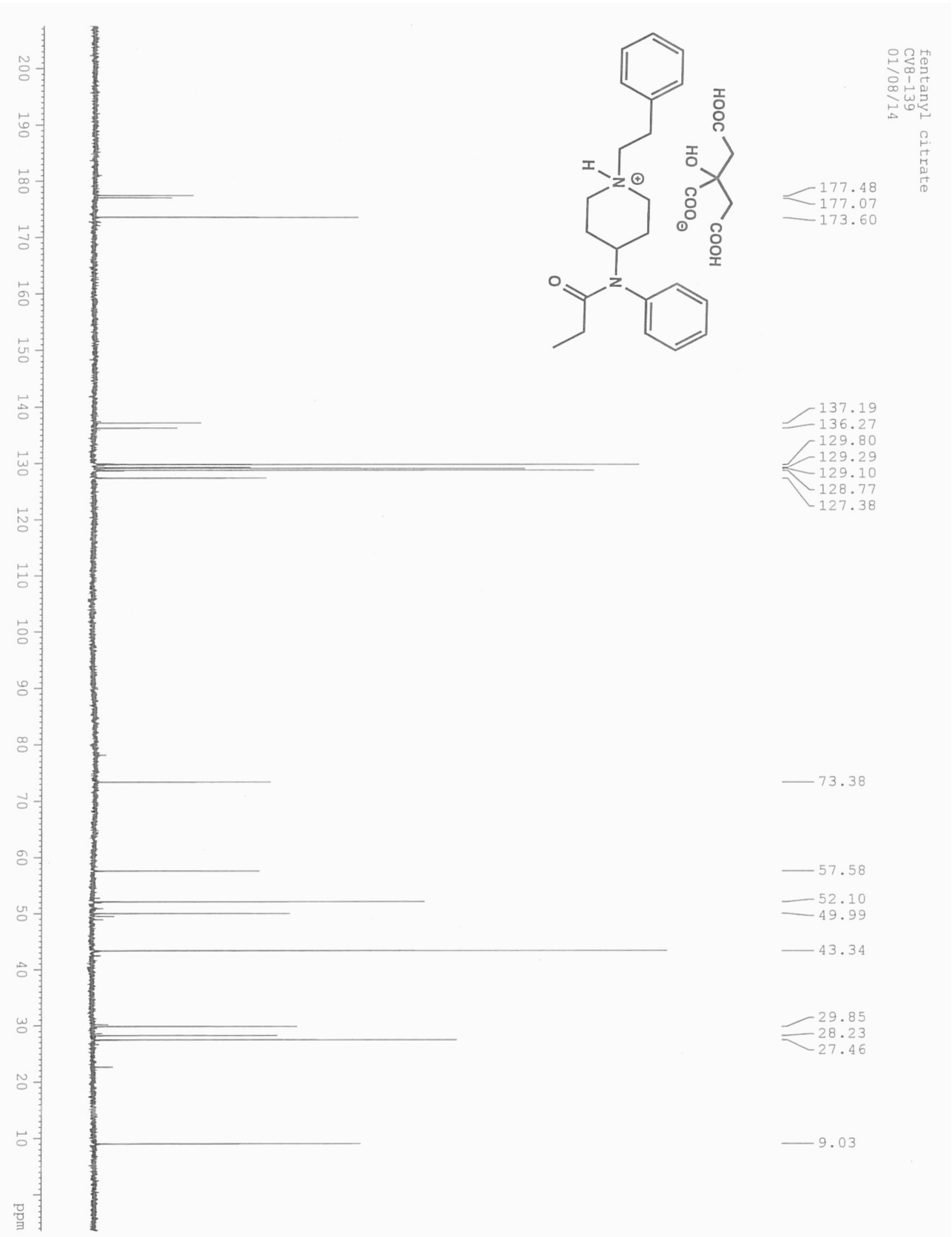
fentanyl citrate
CV8-139
01/08/14
177.48
177.07
173.60
137.19
136.27
129.80
129.29
129.10
128.77
127.38
73.38
57.58
52.10
49.99
43.34
29.85
28.23
27.46
9.03
HOOC
HO
COO
COOH
N
H
O
ppm

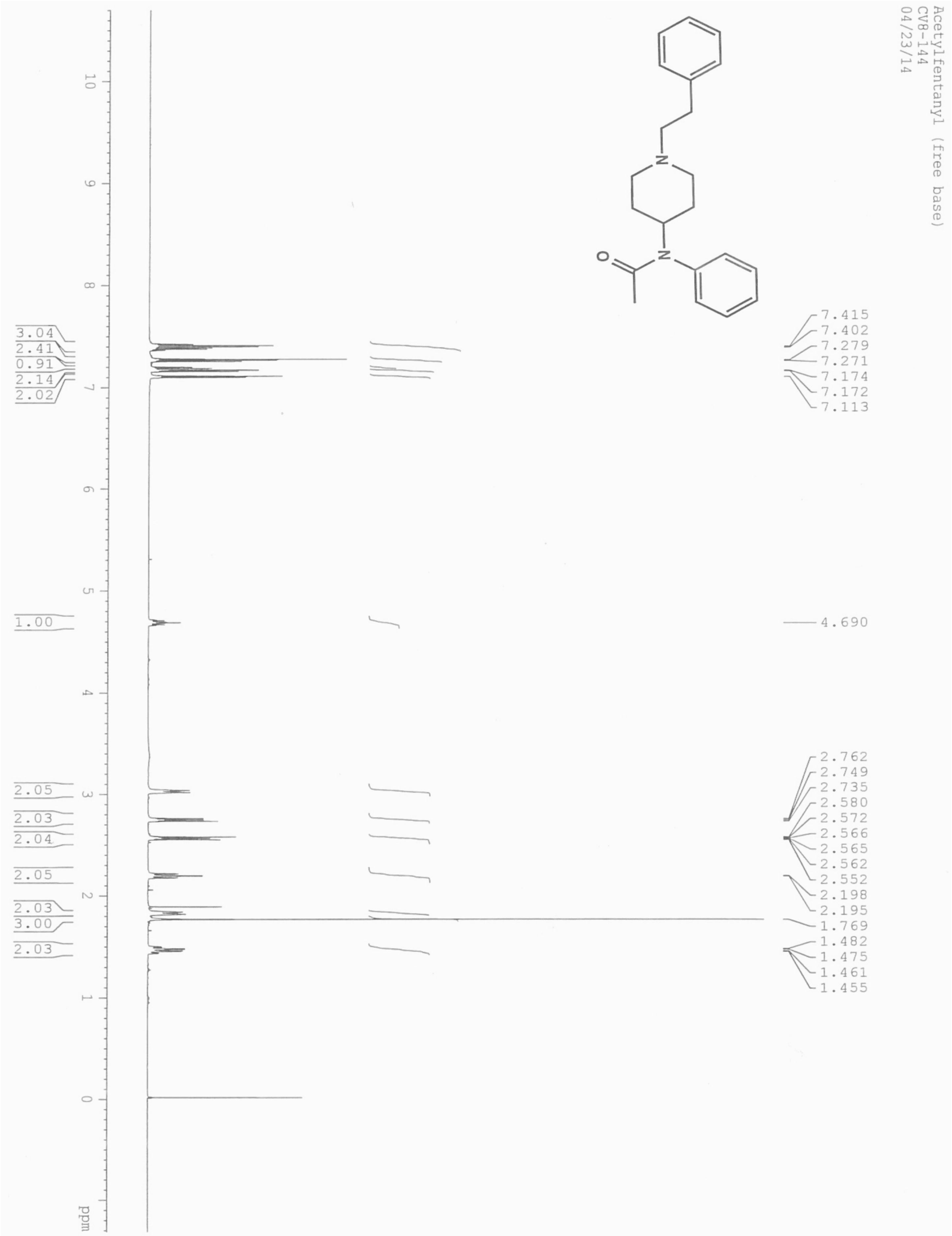
Acetylfentanyl (free base)
CV8-144
04/23/14
7.415
7.402
7.279
7.271
7.174
7.172
7.113
4.690
2.762
2.749
2.735
2.580
2.572
2.566
2.565
2.562
2.552
2.198
2.195
1.769
1.482
1.475
1.461
1.455
3.04
2.41
0.91
2.14
2.02
1.00
2.05
2.03
2.04
2.05
2.03
3.00
2.03
ppm

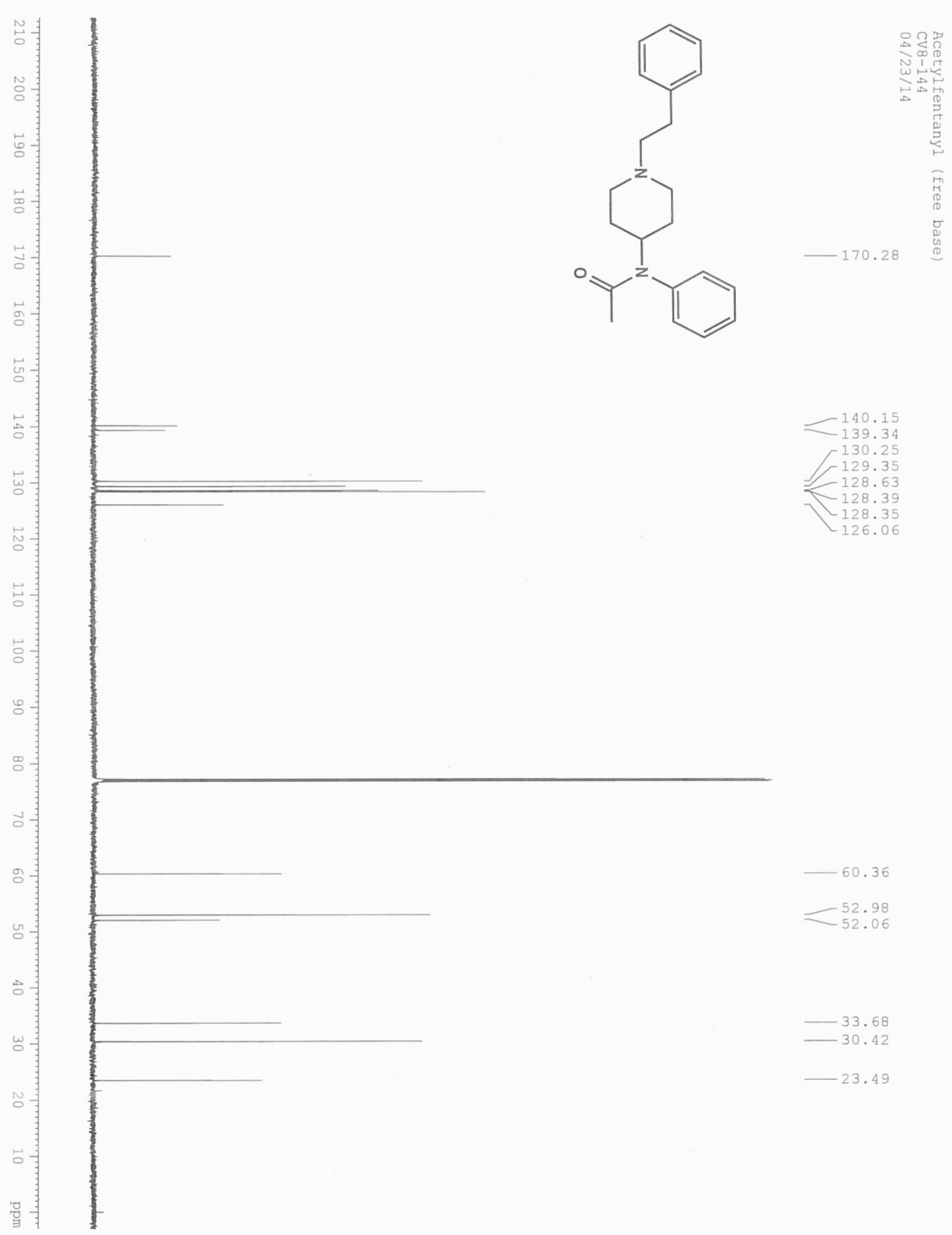
Acetylfentanyl (free base)
CV8-144
04/23/14
170.28
140.15
139.34
130.25
129.35
128.63
128.39
128.35
126.06
60.36
52.98
52.06
33.68
30.42
23.49
210
200
190
180
170
160
150
140
130
120
110
100
90
80
70
60
50
40
30
20
10
ppm
N
N
O

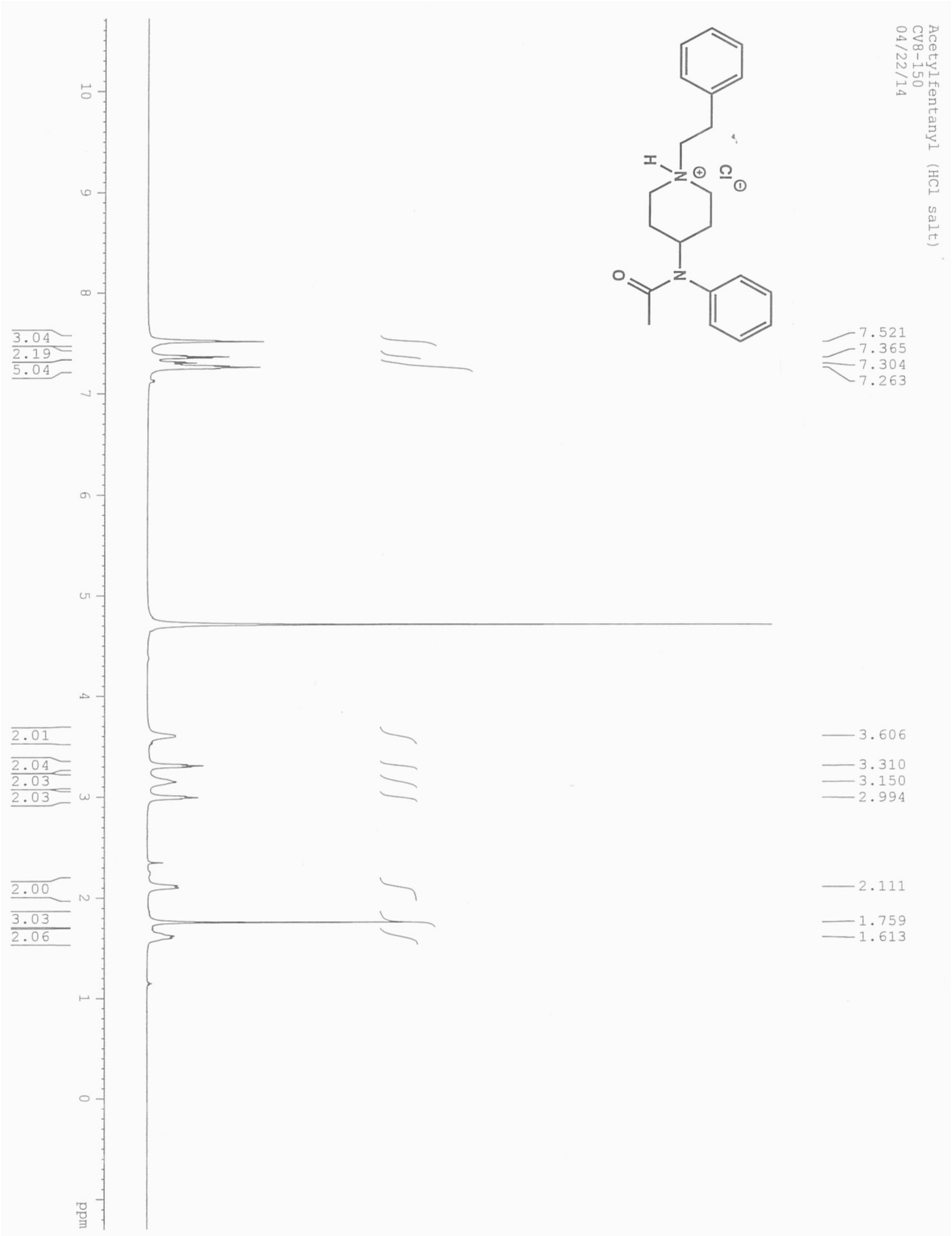
Acetylfentanyl (HCl salt)
CV8-150
04/22/14
7.521
7.365
7.304
7.263
3.606
3.310
3.150
2.994
2.111
1.759
1.613
3.04
2.19
5.04
2.01
2.04
2.03
2.03
2.00
3.03
2.06
ppm

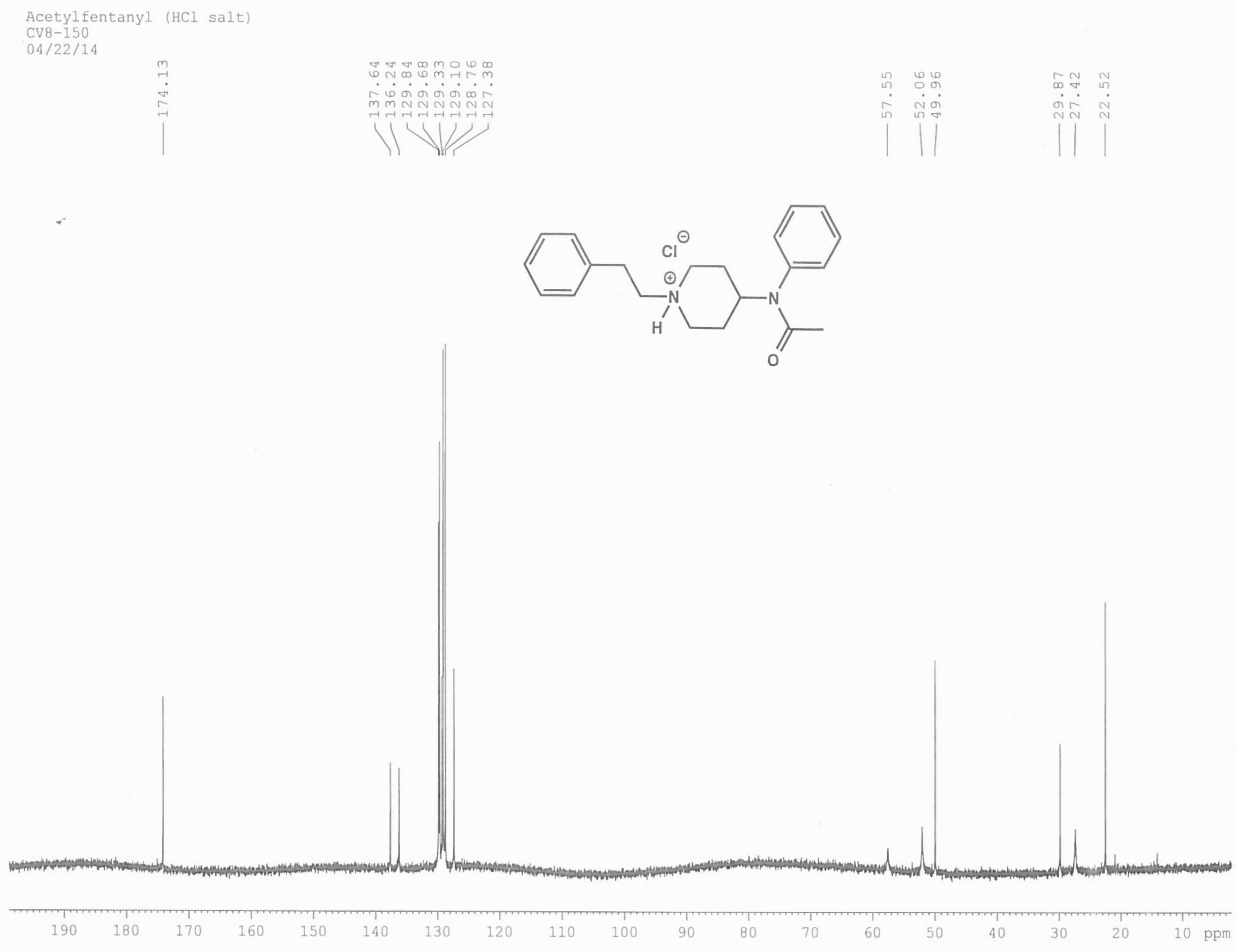
Acetylfentanyl (HCl salt)
CV8-150
04/22/14
174.13
137.64
136.24
129.84
129.68
129.33
129.10
128.76
127.38
57.55
52.06
49.96
29.87
27.42
22.52
Cl
N
H
N
O
190 180 170 160 150 140 130 120 110 100 90 80 70 60 50 40 30 20 10 ppm

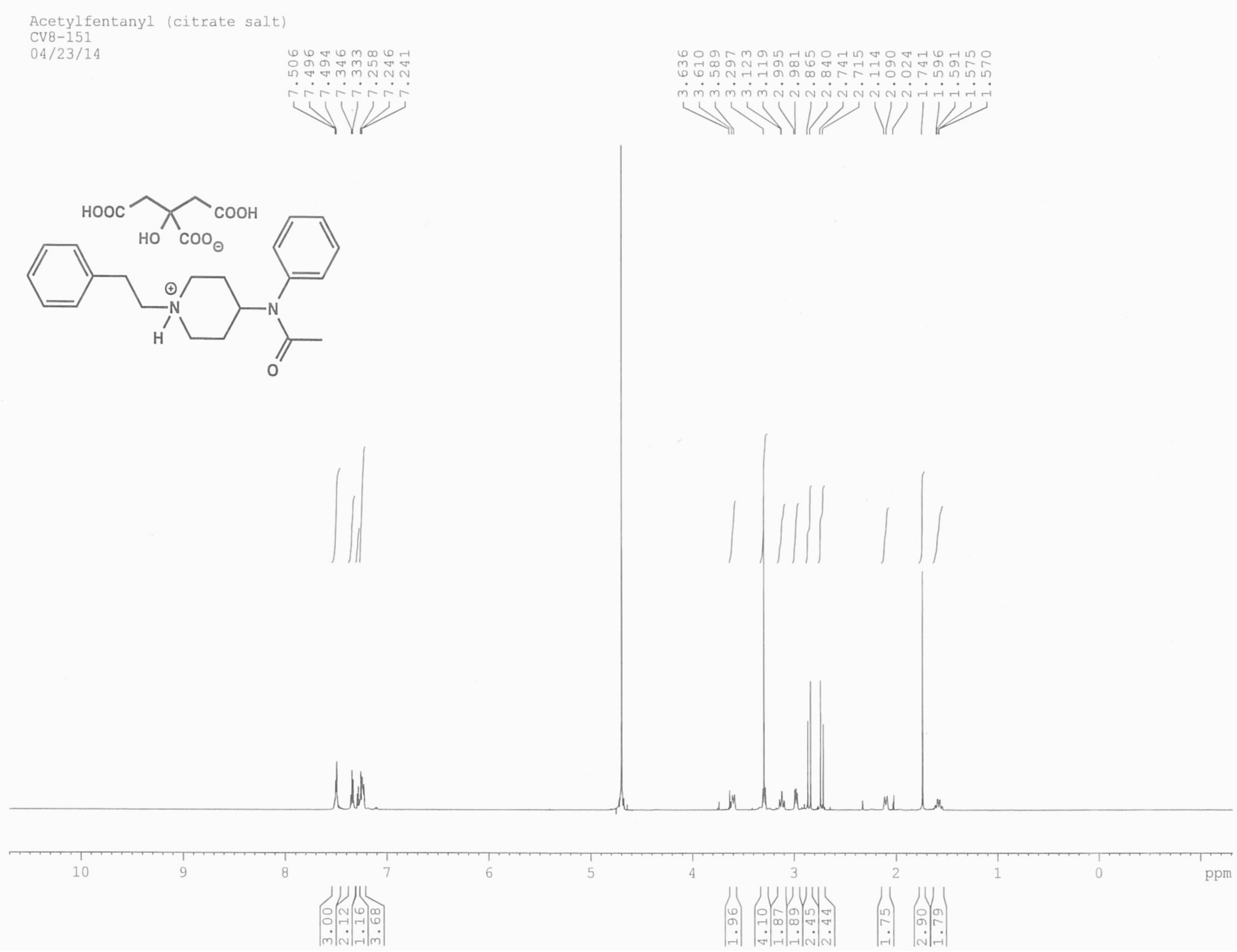
Acetylfentanyl (citrate salt)
CV8-151
04/23/14
HOOC
COOH
HO
COO
N
H
N
O
7.506
7.496
7.494
7.346
7.333
7.258
7.246
7.241
3.636
3.610
3.589
3.297
3.123
3.119
2.995
2.981
2.865
2.840
2.741
2.715
2.114
2.090
2.024
1.741
1.596
1.591
1.575
1.570
10
9
8
7
6
5
4
3
2
1
0
ppm
3.00
2.12
1.16
3.68
1.96
4.10
1.87
1.89
2.45
2.44
1.75
2.90
1.79

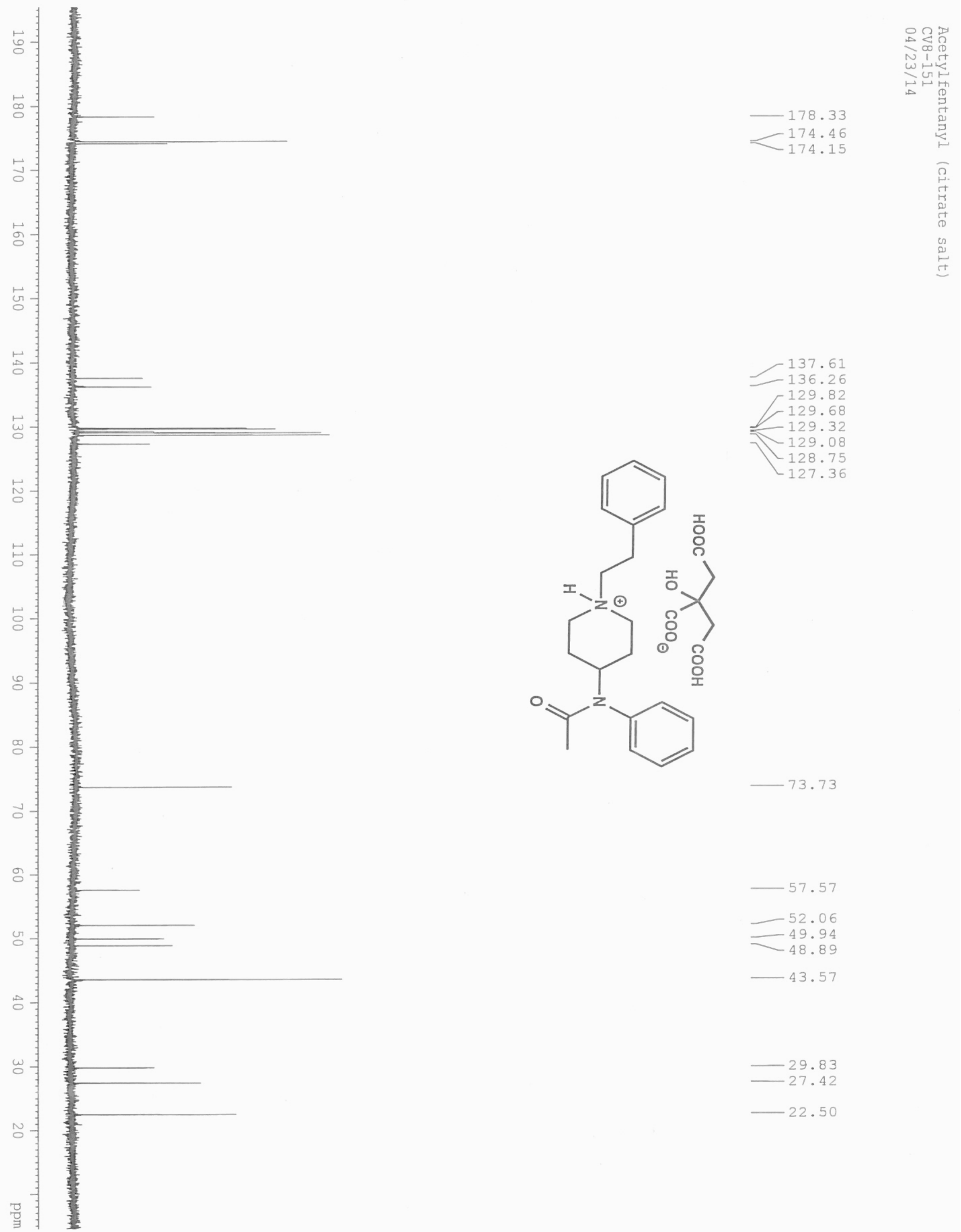
Acetylfentanyl (citrate salt)
CV8-151
04/23/14
178.33
174.46
174.15
137.61
136.26
129.82
129.68
129.32
129.08
128.75
127.36
73.73
57.57
52.06
49.94
48.89
43.57
29.83
27.42
22.50
190
180
170
160
150
140
130
120
110
100
90
80
70
60
50
40
30
20
ppm
HOOC
HO
COO
COOH
H
N
O

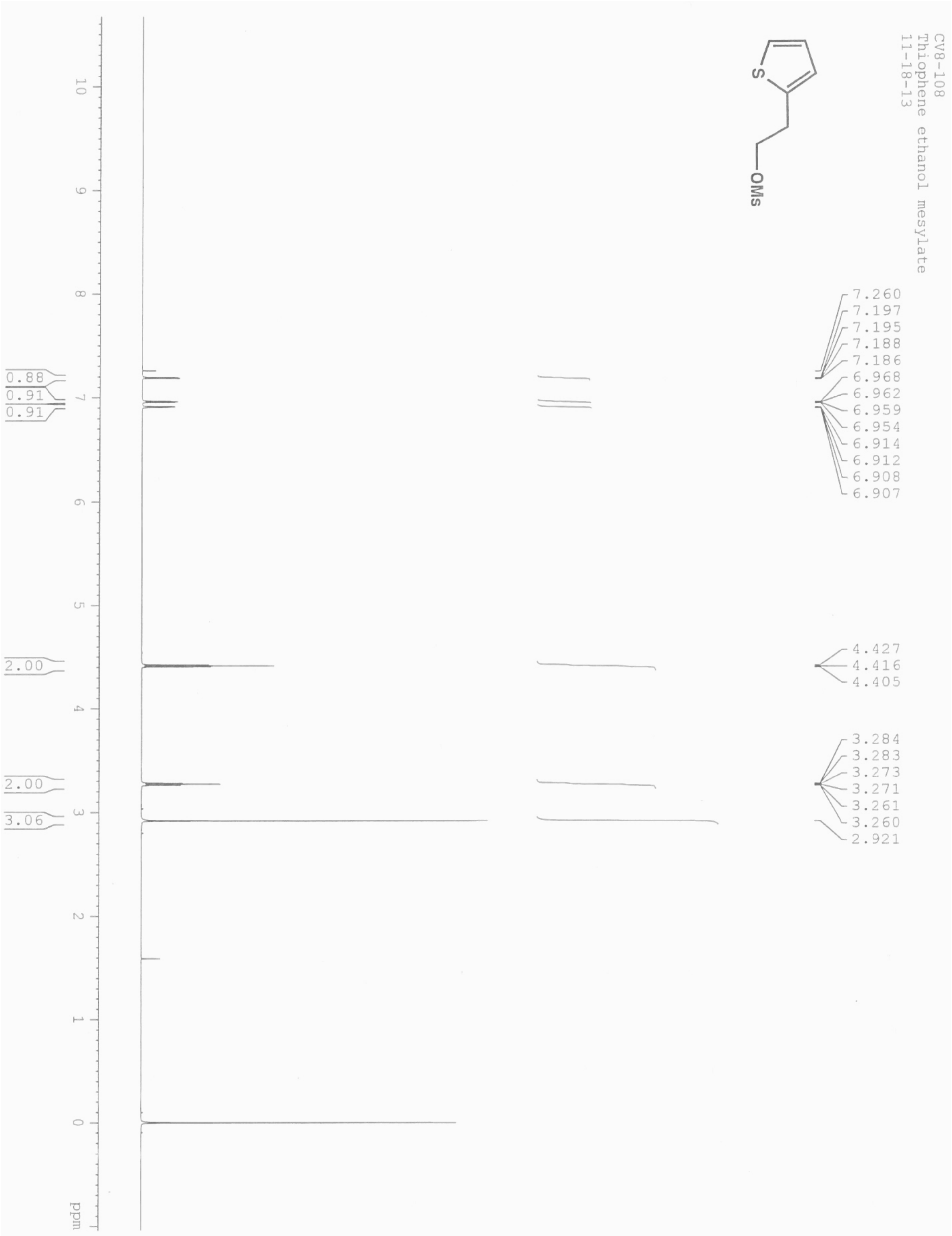
CV8-108
Thiophene ethanol mesylate
11-18-13
S
OMs
7.260
7.197
7.195
7.188
7.186
6.968
6.962
6.959
6.954
6.914
6.912
6.908
6.907
4.427
4.416
4.405
3.284
3.283
3.273
3.271
3.261
3.260
2.921
0.88
0.91
0.91
2.00
2.00
3.06
ppm

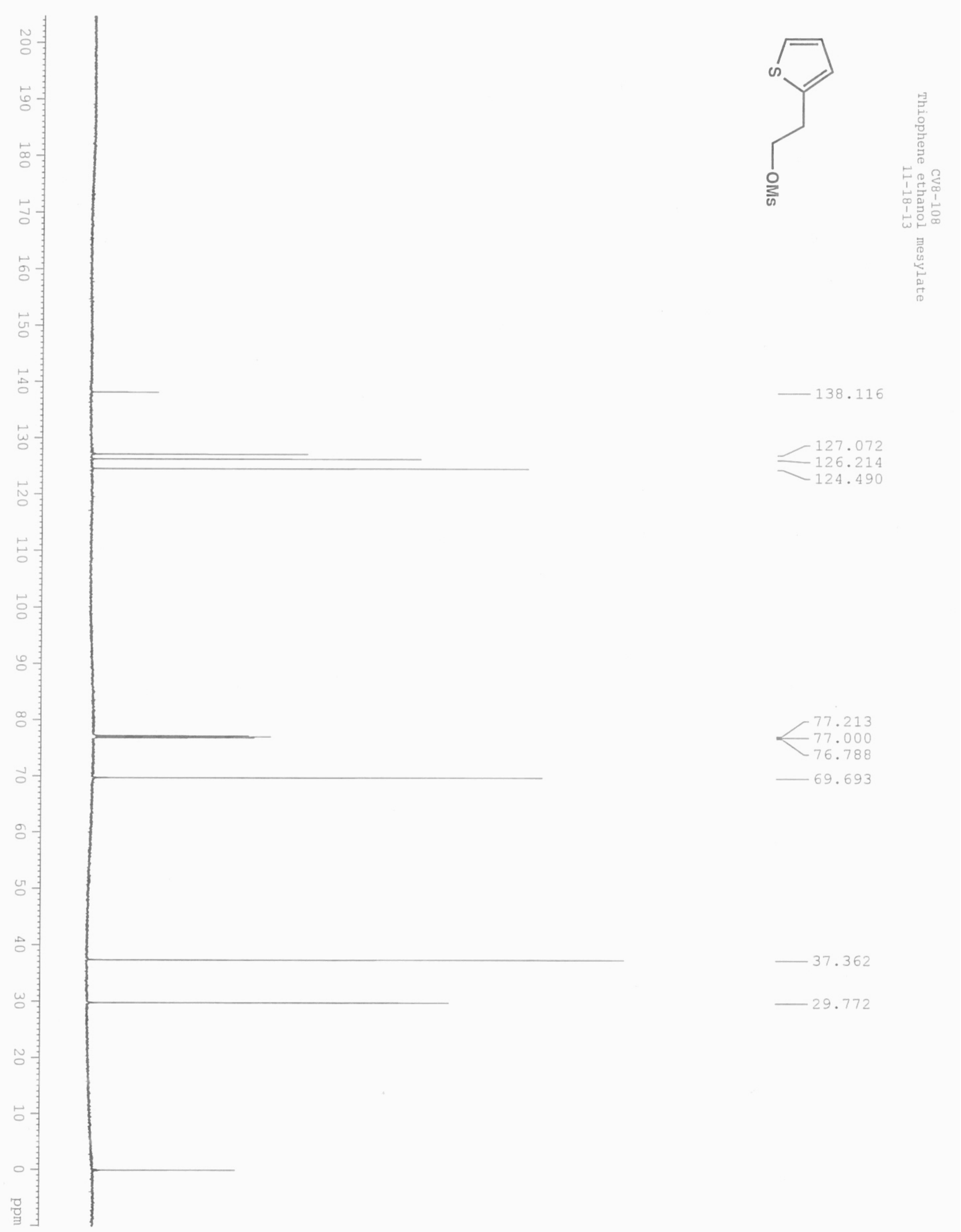
CV8-108
Thiophene ethanol mesylate
11-18-13
S
OMs
138.116
127.072
126.214
124.490
77.213
77.000
76.788
69.693
37.362
29.772
200
190
180
170
160
150
140
130
120
110
100
90
80
70
60
50
40
30
20
10
0
ppm

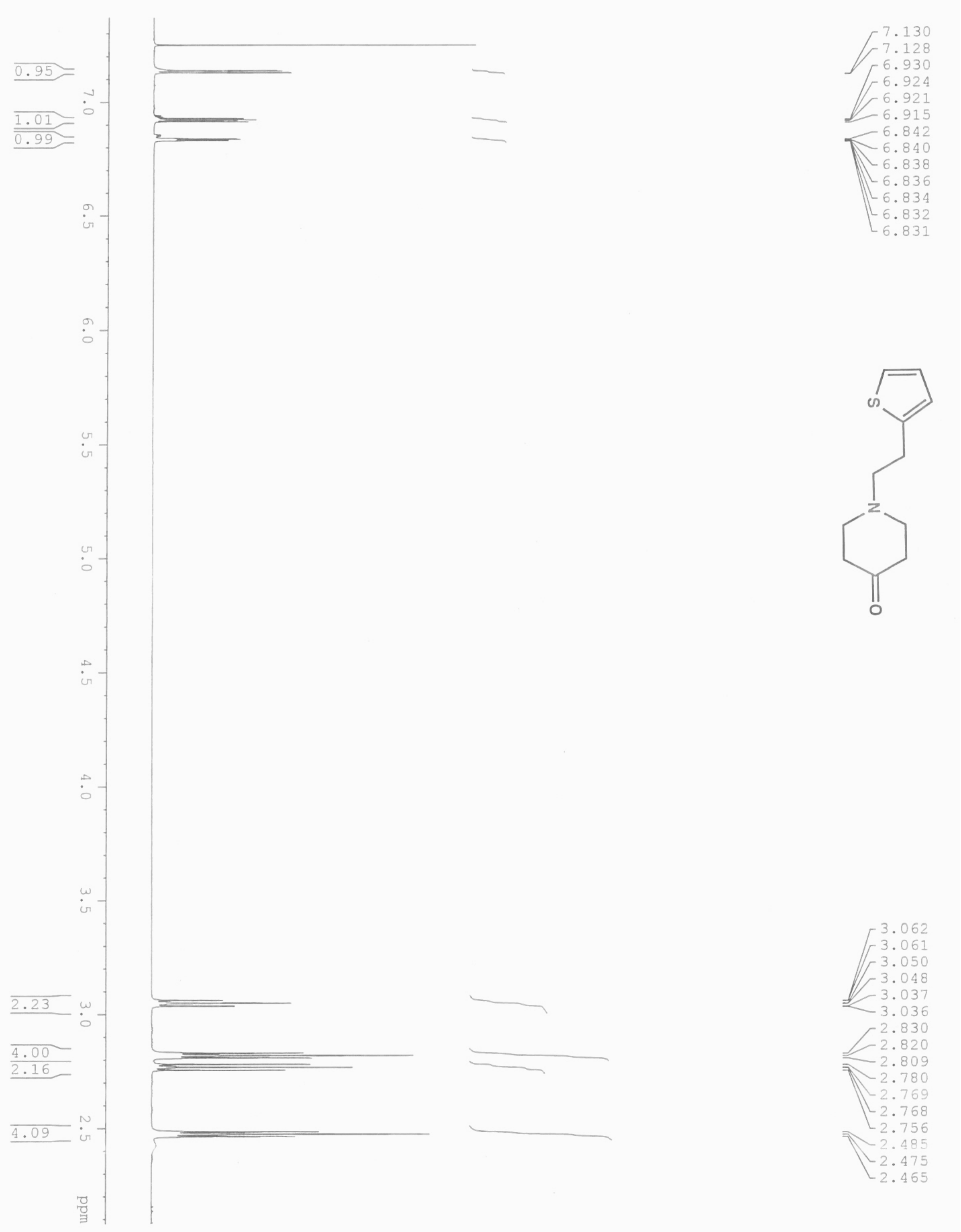
7.130
7.128
6.930
6.924
6.921
6.915
6.842
6.840
6.838
6.836
6.834
6.832
6.831
3.062
3.061
3.050
3.048
3.037
3.036
2.830
2.820
2.809
2.780
2.769
2.768
2.756
2.485
2.475
2.465
0.95
1.01
0.99
2.23
4.00
2.16
4.09
7.0
6.5
6.0
5.5
5.0
4.5
4.0
3.5
3.0
2.5
ppm
S
N
O

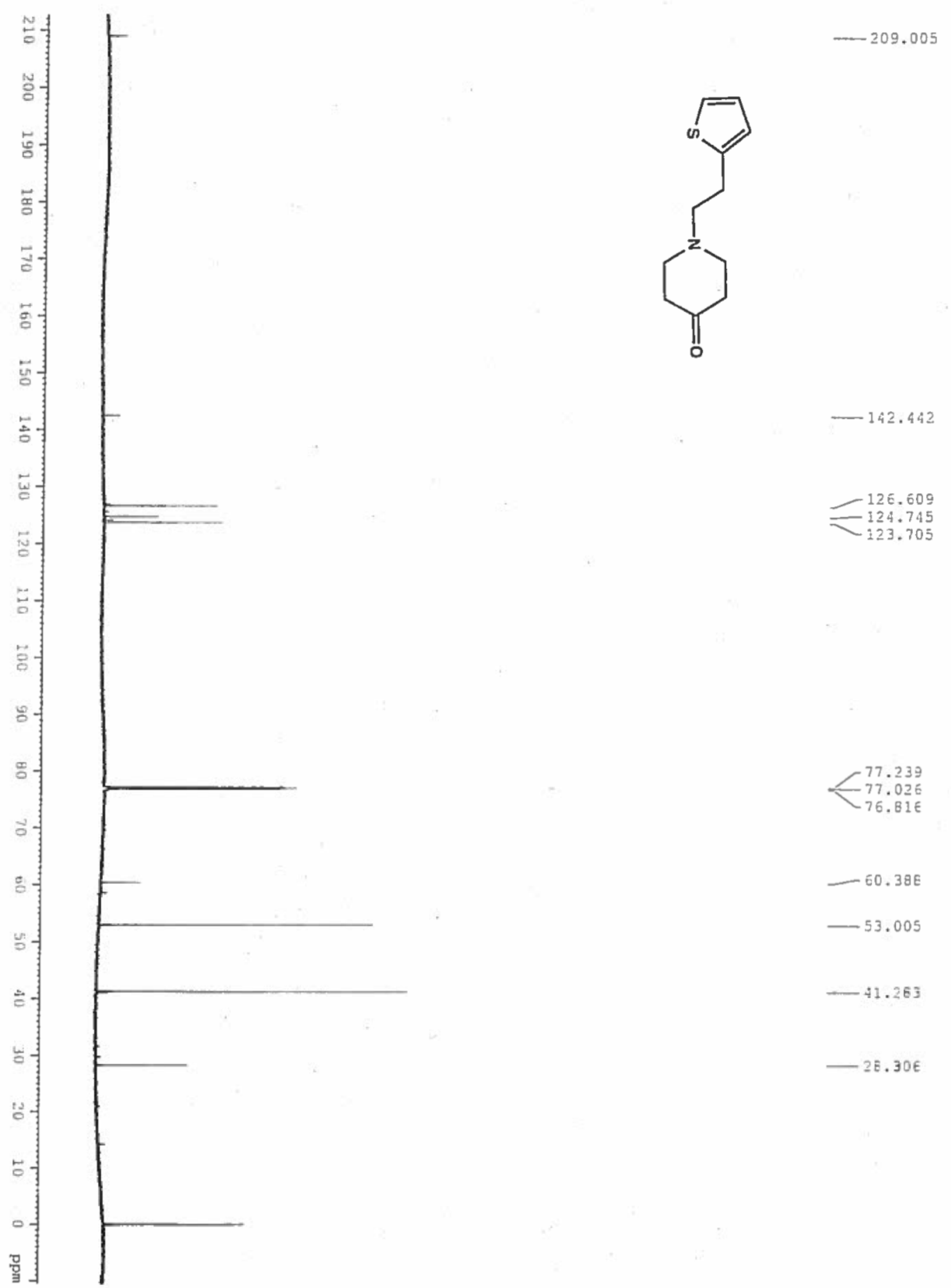
209.005
142.442
126.609
124.745
123.705
77.239
77.026
76.816
60.388
53.005
41.263
28.306
ppm
S
N
O

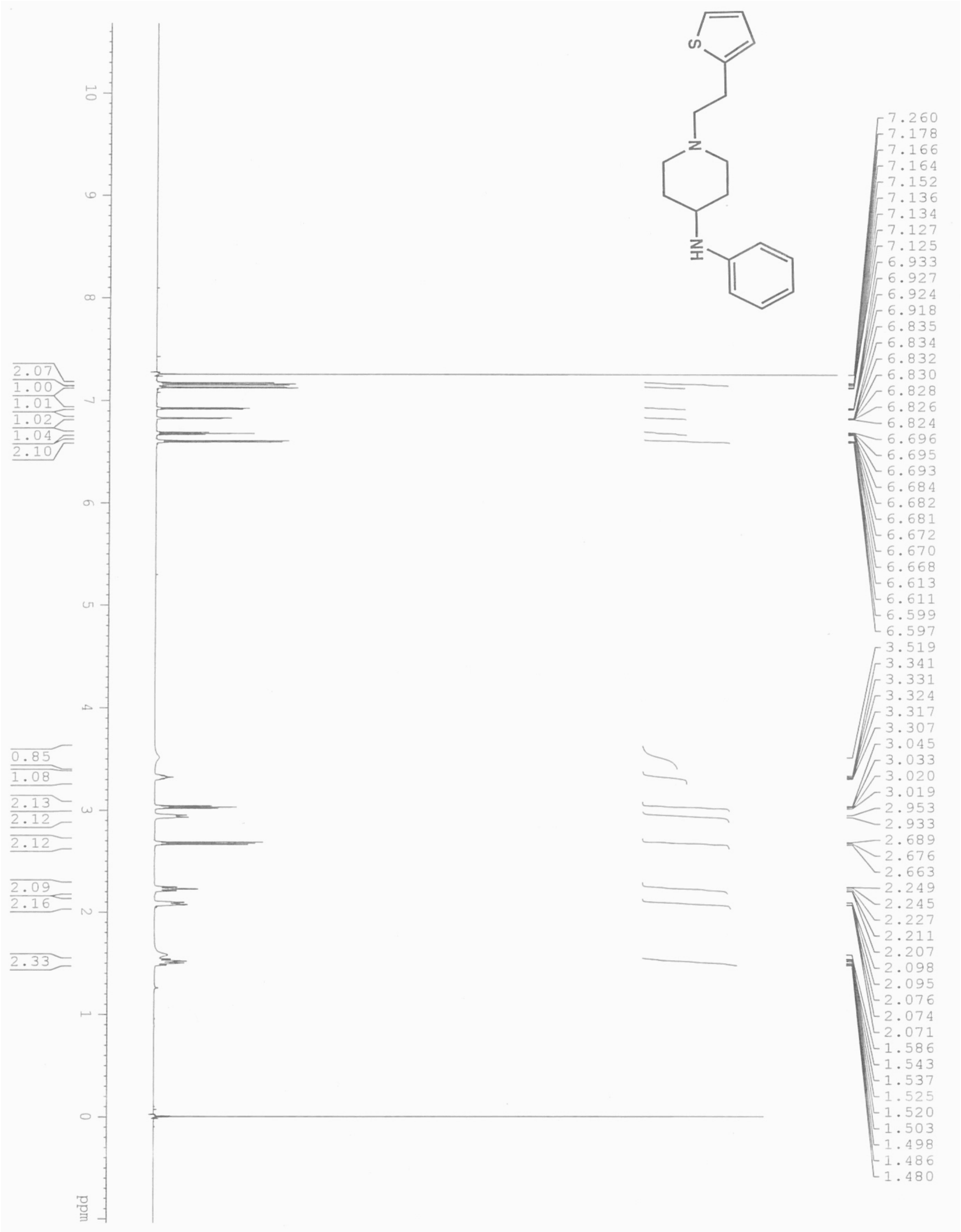
7.260
7.178
7.166
7.164
7.152
7.136
7.134
7.127
7.125
6.933
6.927
6.924
6.918
6.835
6.834
6.832
6.830
6.828
6.826
6.824
6.696
6.695
6.693
6.684
6.682
6.681
6.672
6.670
6.668
6.613
6.611
6.599
6.597
3.519
3.341
3.331
3.324
3.317
3.307
3.045
3.033
3.020
3.019
2.953
2.933
2.689
2.676
2.663
2.249
2.245
2.227
2.211
2.207
2.098
2.095
2.076
2.074
2.071
1.586
1.543
1.537
1.525
1.520
1.503
1.498
1.486
1.480
2.07
1.00
1.01
1.02
1.04
2.10
0.85
1.08
2.13
2.12
2.12
2.09
2.16
2.33
ppm
S
N
NH

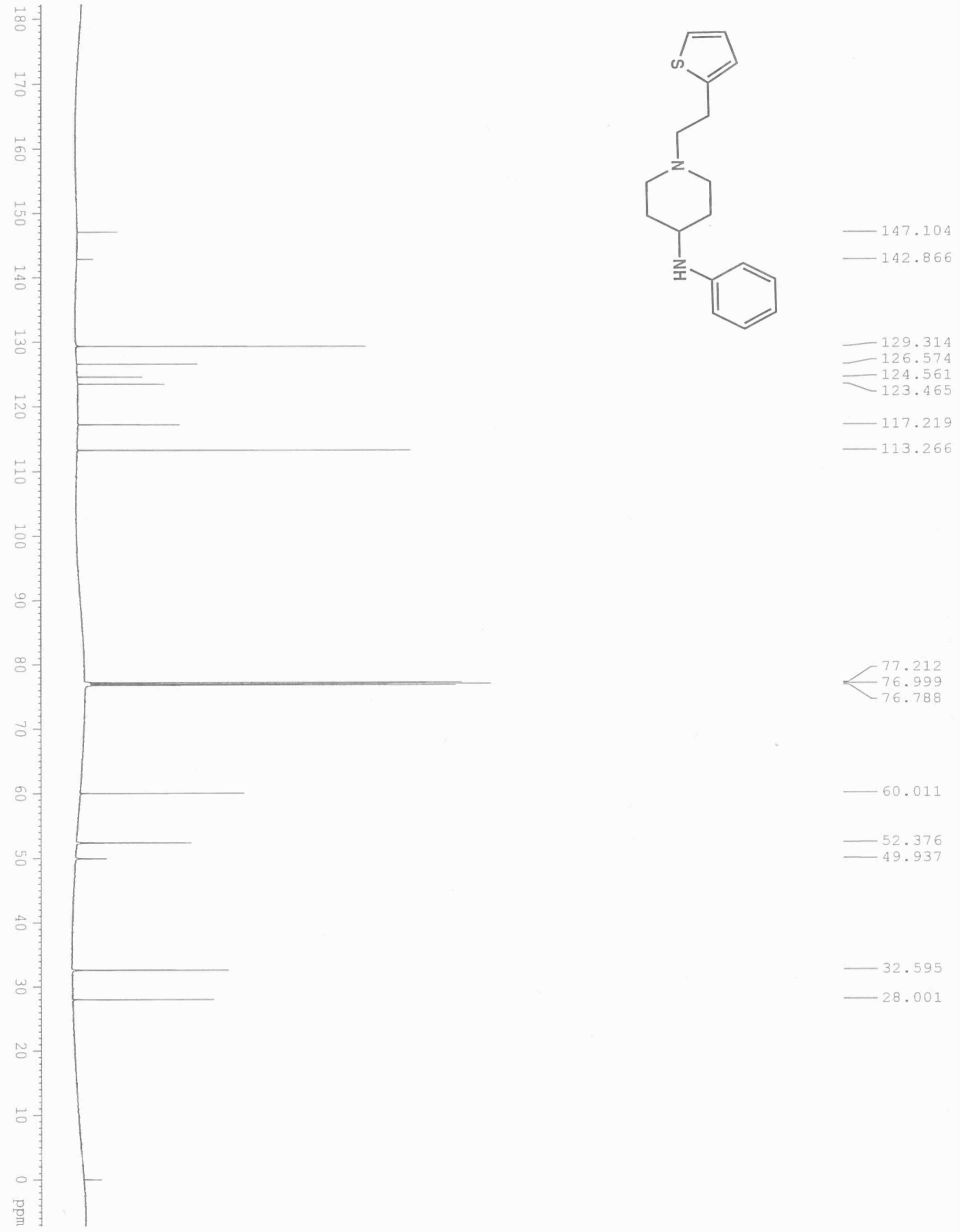
S
N
NH
147.104
142.866
129.314
126.574
124.561
123.465
117.219
113.266
77.212
76.999
76.788
60.011
52.376
49.937
32.595
28.001
180
170
160
150
140
130
120
110
100
90
80
70
60
50
40
30
20
10
0
ppm

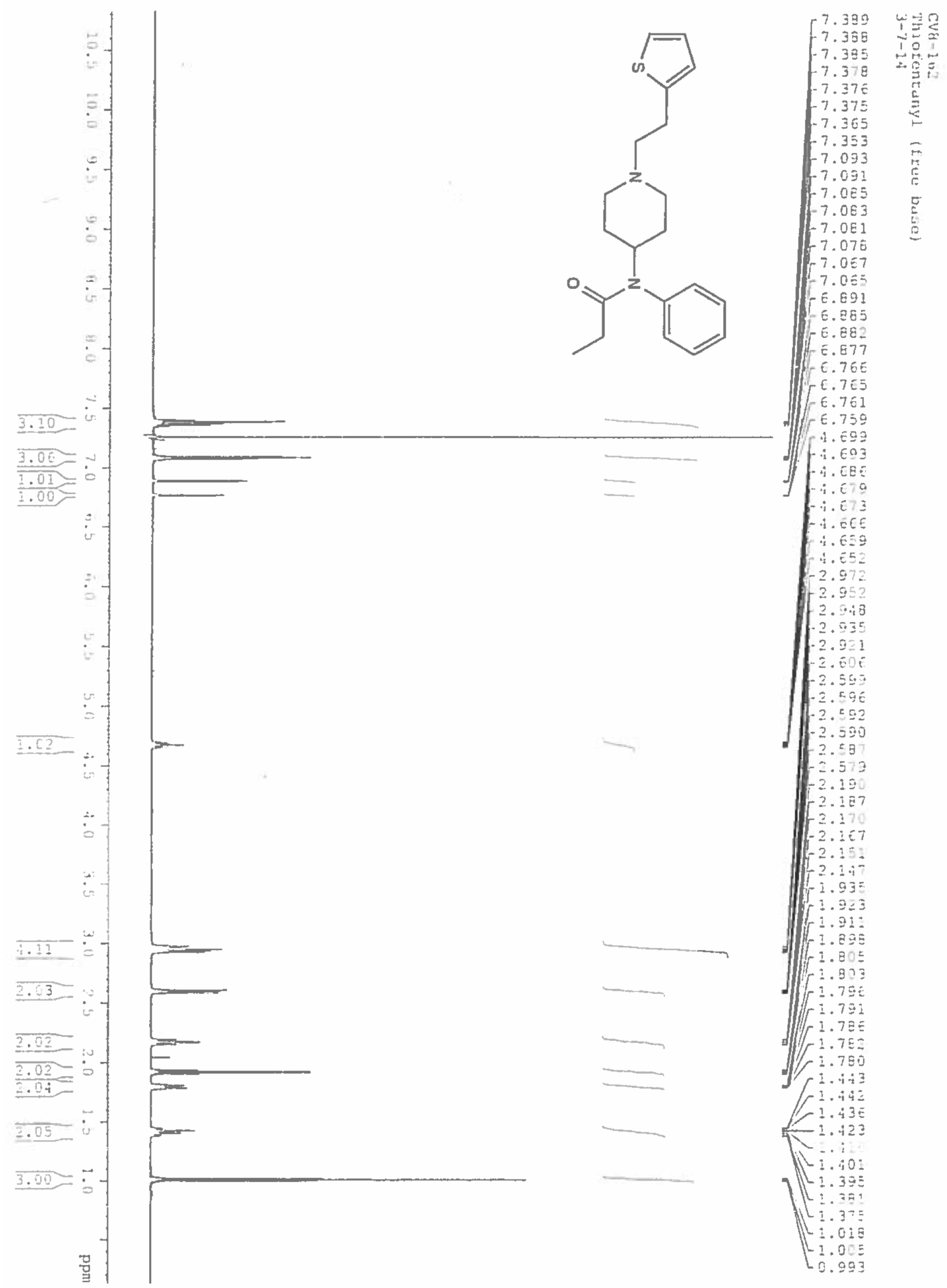
CV8-162
Thiofentanyl (free base)
3-7-14

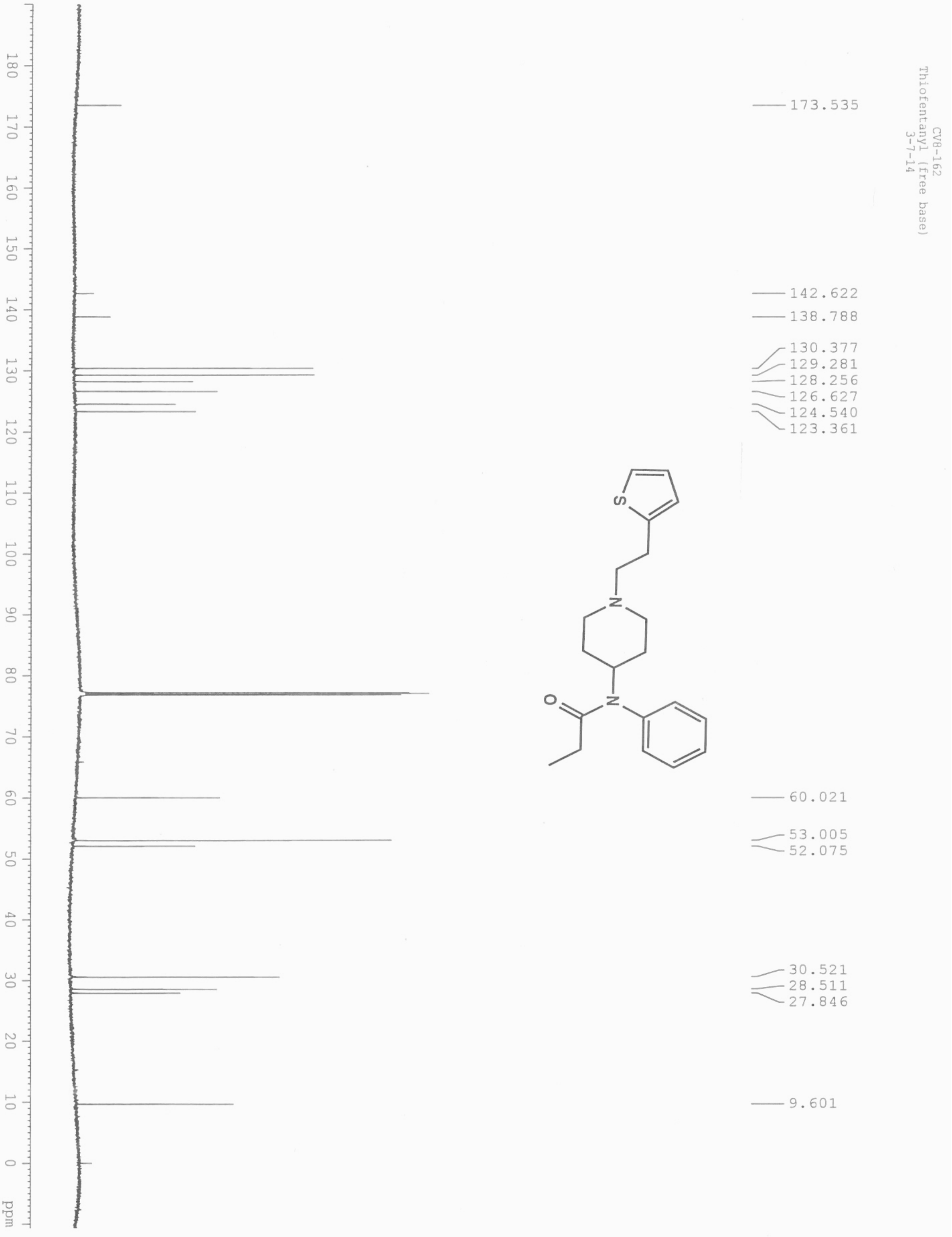
CV8-162
Thiofentanyl (free base)
3-7-14
173.535
142.622
138.788
130.377
129.281
128.256
126.627
124.540
123.361
60.021
53.005
52.075
30.521
28.511
27.846
9.601
S
N
N
O
ppm

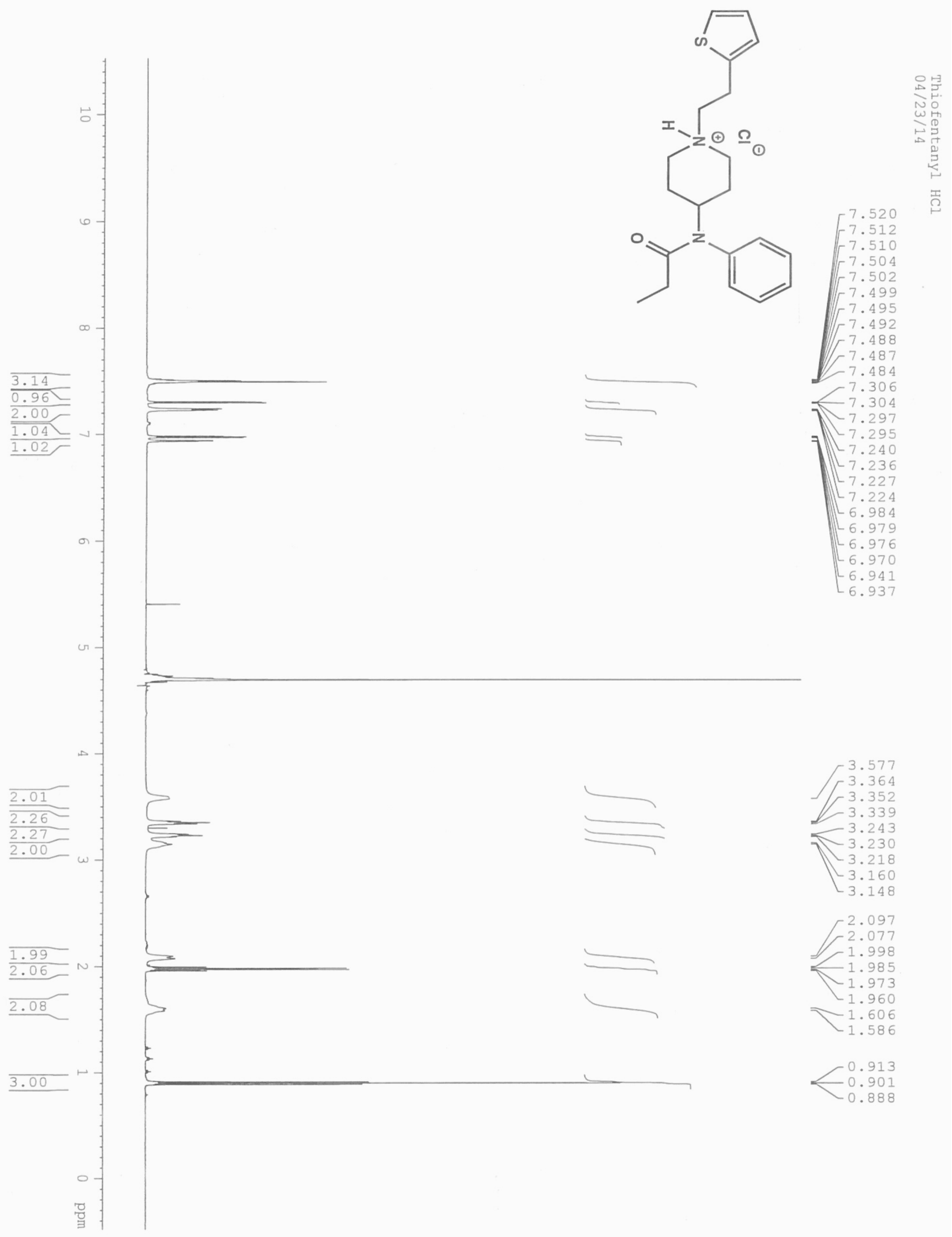
Thiofentanyl HCl
04/23/14
7.520
7.512
7.510
7.504
7.502
7.499
7.495
7.492
7.488
7.487
7.484
7.306
7.304
7.297
7.295
7.240
7.236
7.227
7.224
6.984
6.979
6.976
6.970
6.941
6.937
3.577
3.364
3.352
3.339
3.243
3.230
3.218
3.160
3.148
2.097
2.077
1.998
1.985
1.973
1.960
1.606
1.586
0.913
0.901
0.888
3.14
0.96
2.00
1.04
1.02
2.01
2.26
2.27
2.00
1.99
2.06
2.08
3.00
ppm

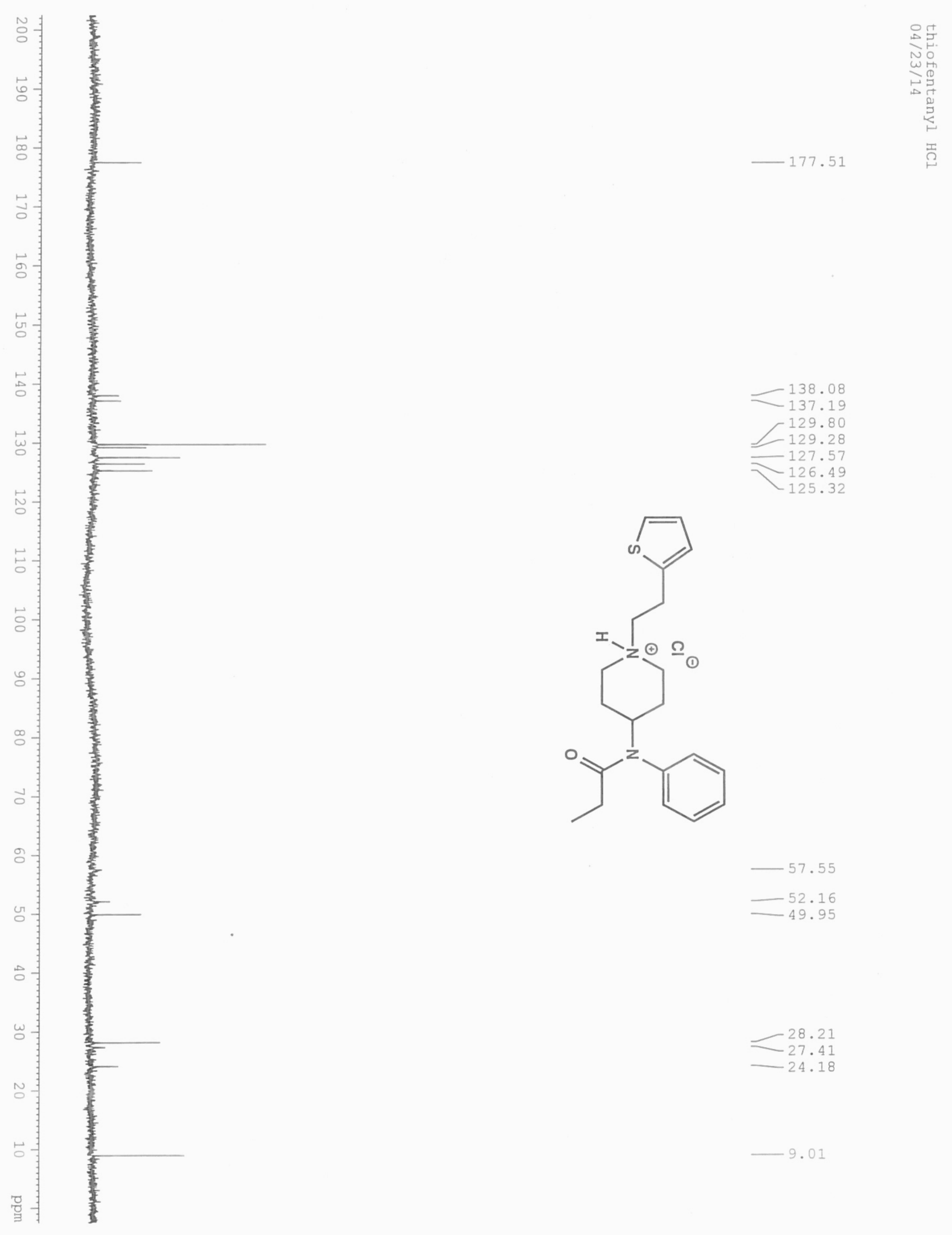
thiofentanyl HCl
04/23/14
177.51
138.08
137.19
129.80
129.28
127.57
126.49
125.32
57.55
52.16
49.95
28.21
27.41
24.18
9.01
S
N
H
Cl
O
ppm

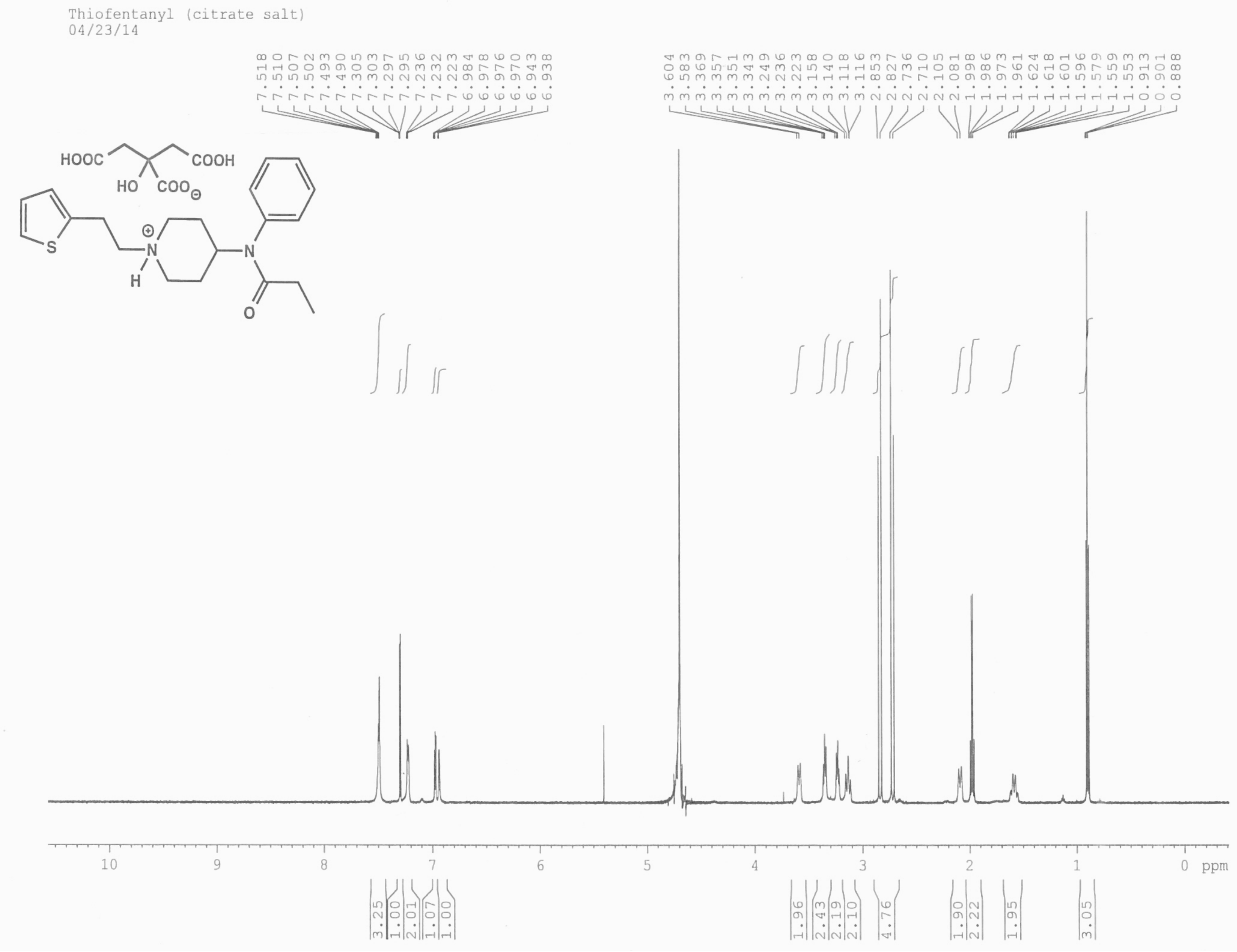
Thiofentanyl (citrate salt)
04/23/14
7.518 7.510 7.507 7.502 7.493 7.490 7.305 7.303 7.297 7.295 7.236 7.232 7.223 6.984 6.978 6.976 6.970 6.943 6.938
3.604 3.583 3.369 3.357 3.351 3.343 3.249 3.236 3.223 3.158 3.140 3.118 3.116 2.853 2.827 2.736 2.710 2.105 2.081 1.998 1.986 1.973 1.961 1.624 1.618 1.601 1.596 1.579 1.559 1.553 0.913 0.901 0.888
HOOC
COOH
HO
COO
S
N
H
N
O
10
9
8
7
6
5
4
3
2
1
0
ppm
3.25
1.00
2.01
1.07
1.00
1.96
2.43
2.19
2.10
4.76
1.90
2.22
1.95
3.05

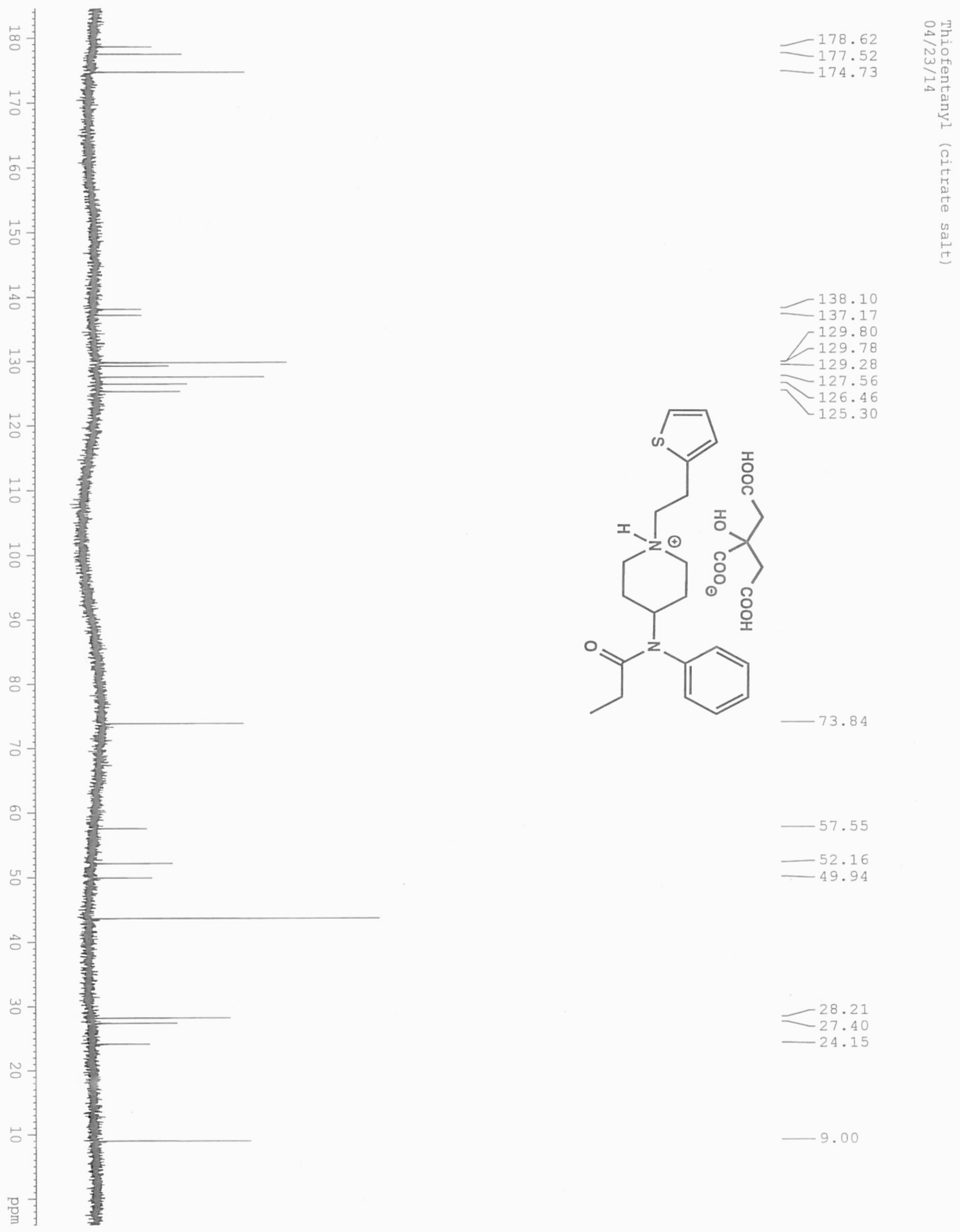
Thiofentanyl (citrate salt)
04/23/14
178.62
177.52
174.73
138.10
137.17
129.80
129.78
129.28
127.56
126.46
125.30
73.84
57.55
52.16
49.94
28.21
27.40
24.15
9.00
HOOC
HO
COO
COOH
S
N
H
O
180
170
160
150
140
130
120
110
100
90
80
70
60
50
40
30
20
10
ppm

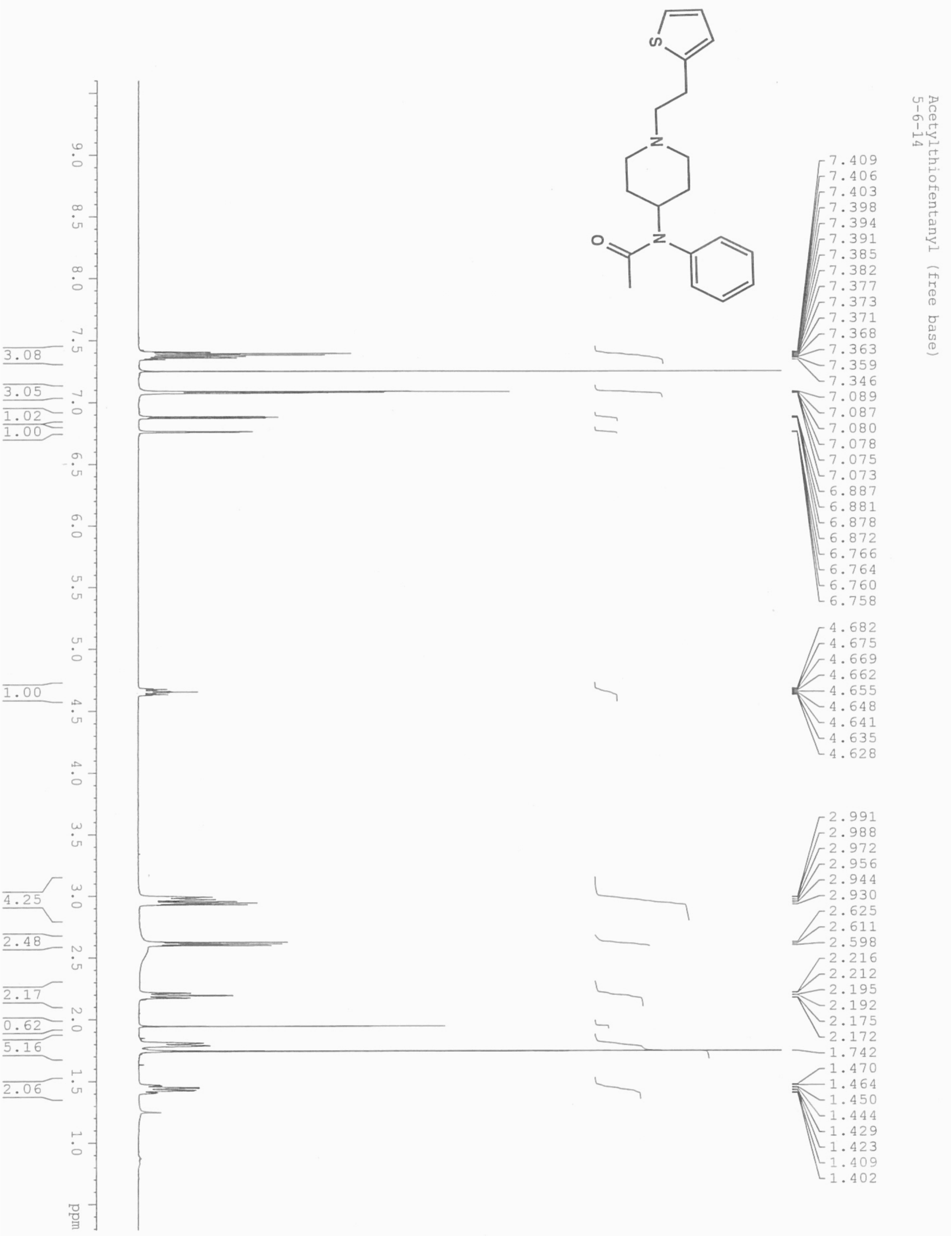
Acetylthiofentanyl (free base)
5-6-14
7.409
7.406
7.403
7.398
7.394
7.391
7.385
7.382
7.377
7.373
7.371
7.368
7.363
7.359
7.346
7.089
7.087
7.080
7.078
7.075
7.073
6.887
6.881
6.878
6.872
6.766
6.764
6.760
6.758
4.682
4.675
4.669
4.662
4.655
4.648
4.641
4.635
4.628
2.991
2.988
2.972
2.956
2.944
2.930
2.625
2.611
2.598
2.216
2.212
2.195
2.192
2.175
2.172
1.742
1.470
1.464
1.450
1.444
1.429
1.423
1.409
1.402
3.08
3.05
1.02
1.00
1.00
4.25
2.48
2.17
0.62
5.16
2.06
9.0
8.5
8.0
7.5
7.0
6.5
6.0
5.5
5.0
4.5
4.0
3.5
3.0
2.5
2.0
1.5
1.0
ppm
S
N
O
N

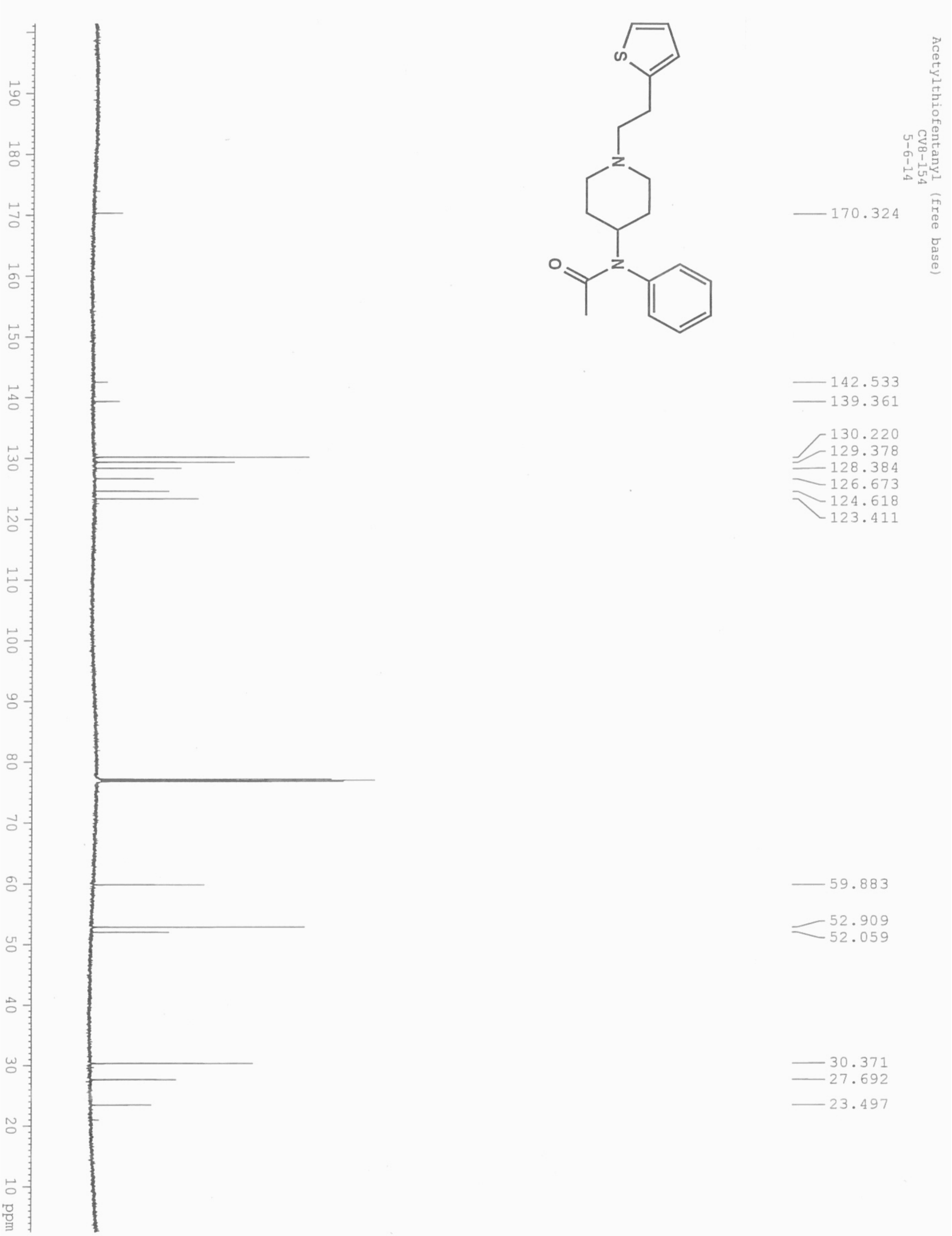
Acetylthiofentanyl (free base)
CV8-154
5-6-14
170.324
142.533
139.361
130.220
129.378
128.384
126.673
124.618
123.411
59.883
52.909
52.059
30.371
27.692
23.497
ppm

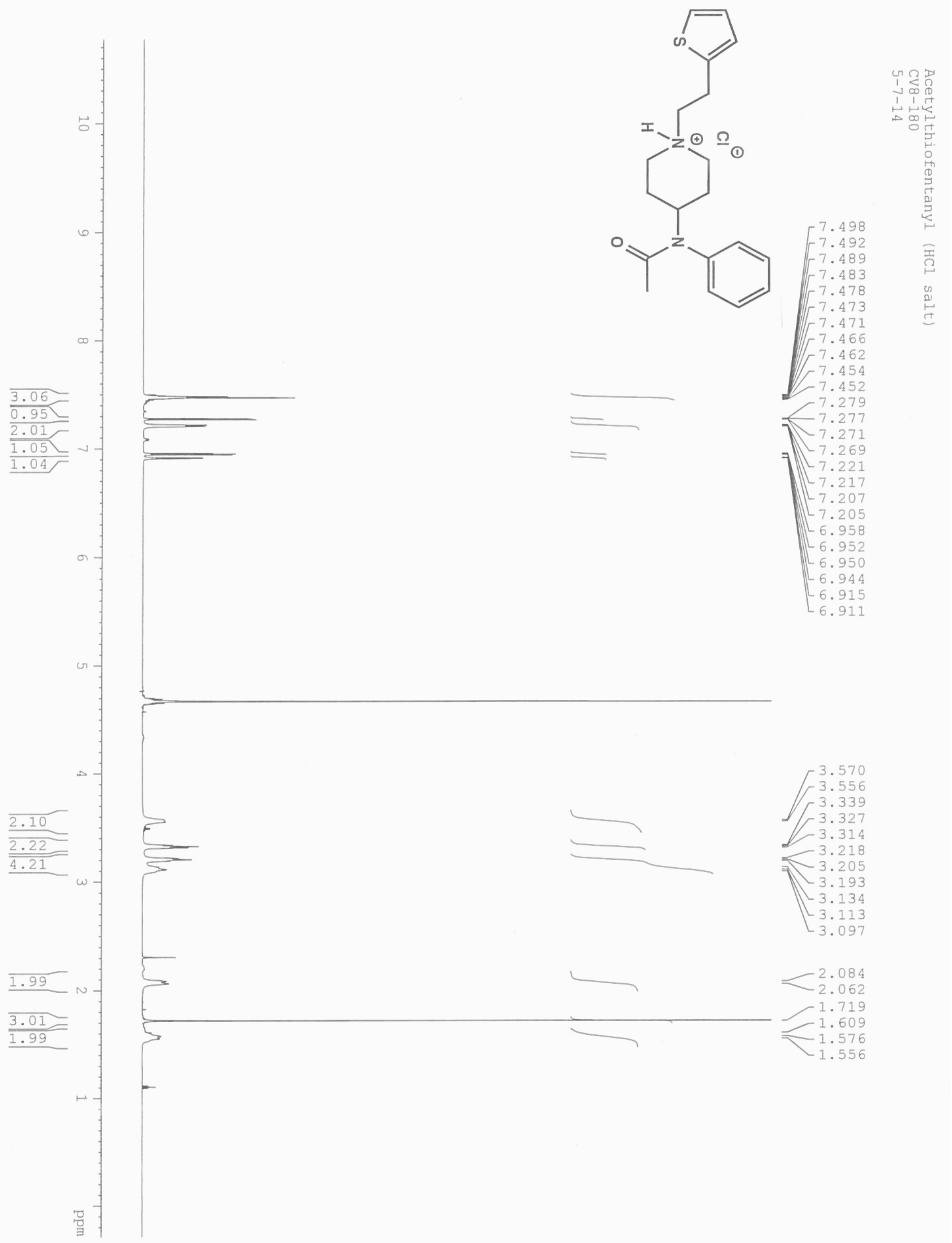
Acetylthiofentanyl (HCl salt)
CV8-180
5-7-14
7.498
7.492
7.489
7.483
7.478
7.473
7.471
7.466
7.462
7.454
7.452
7.279
7.277
7.271
7.269
7.221
7.217
7.207
7.205
6.958
6.952
6.950
6.944
6.915
6.911
3.570
3.556
3.339
3.327
3.314
3.218
3.205
3.193
3.134
3.113
3.097
2.084
2.062
1.719
1.609
1.576
1.556
3.06
0.95
2.01
1.05
1.04
2.10
2.22
4.21
1.99
3.01
1.99
10
9
8
7
6
5
4
3
2
1
ppm

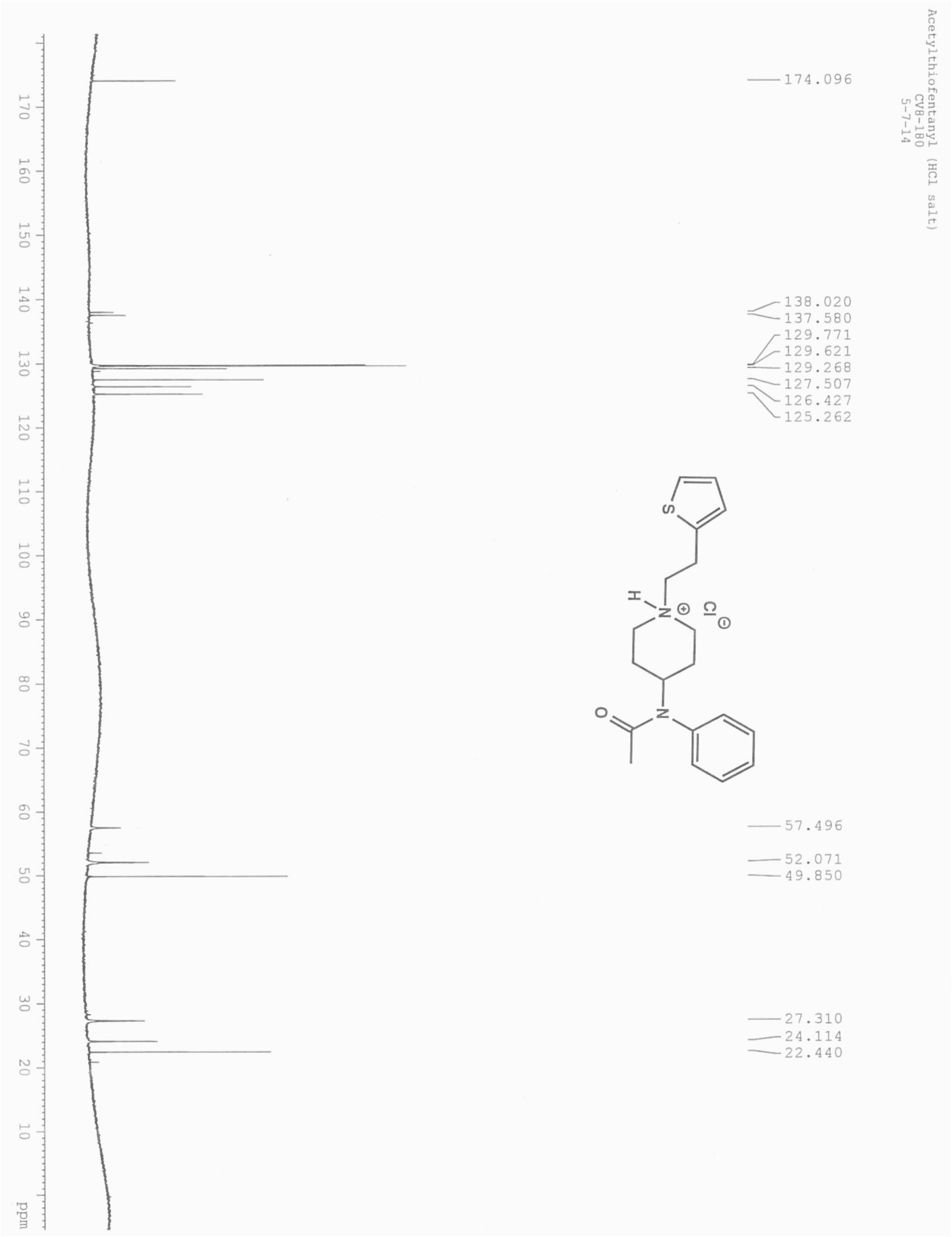

Acetylthiofentanyl (HCl salt)
CV8-180
5-7-14
174.096
138.020
137.580
129.771
129.621
129.268
127.507
126.427
125.262
57.496
52.071
49.850
27.310
24.114
22.440
S
N
H
Cl
O
ppm
170
160
150
140
130
120
110
100
90
80
70
60
50
40
30
20
10

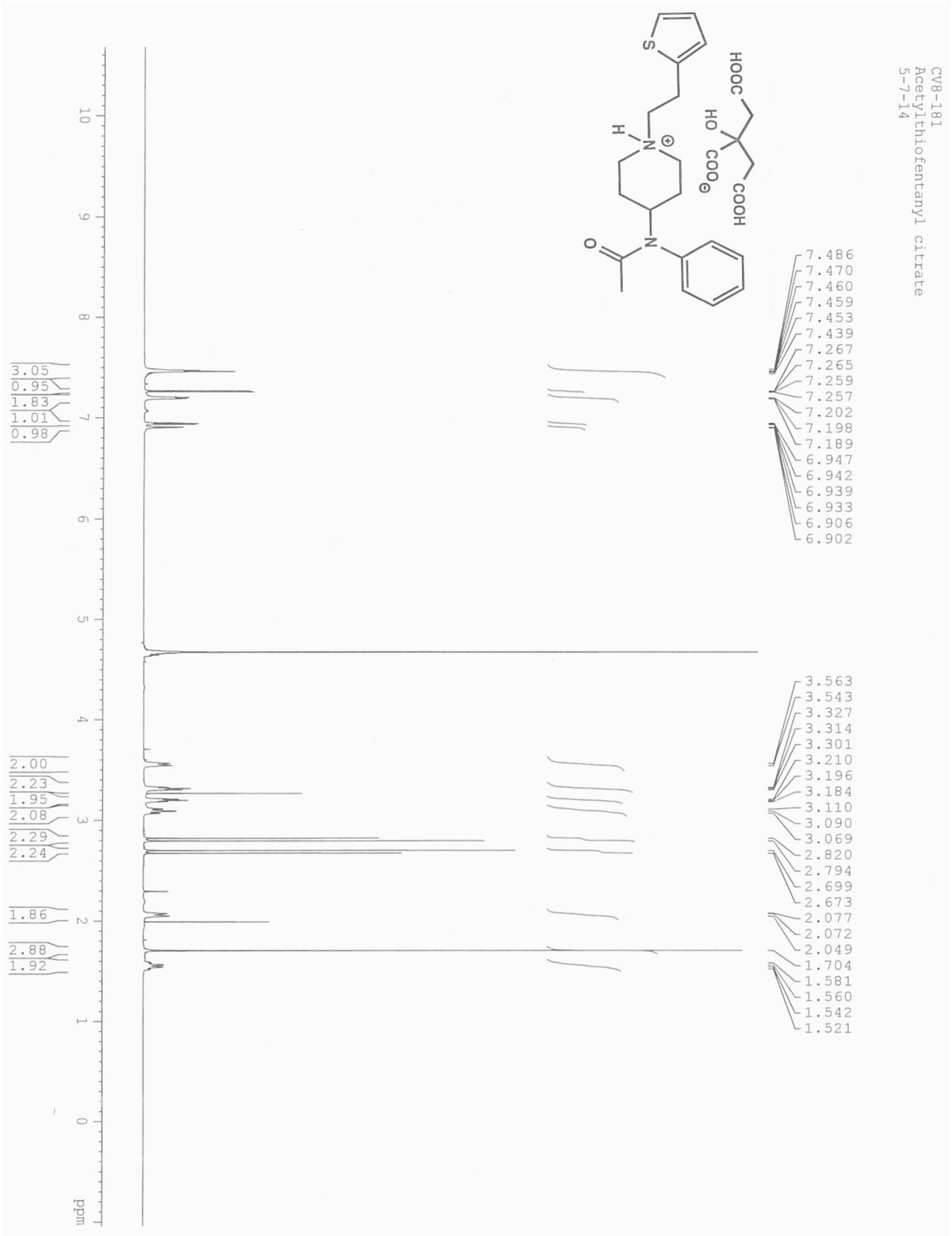
CV8-181
Acetylthiofentanyl citrate
5-7-14
7.486
7.470
7.460
7.459
7.453
7.439
7.267
7.265
7.259
7.257
7.202
7.198
7.189
6.947
6.942
6.939
6.933
6.906
6.902
3.563
3.543
3.327
3.314
3.301
3.210
3.196
3.184
3.110
3.090
3.069
2.820
2.794
2.699
2.673
2.077
2.072
2.049
1.704
1.581
1.560
1.542
1.521
HOOC
COOH
HO
COO
S
N
H
N
O
10
9
8
7
6
5
4
3
2
1
0
ppm
3.05
0.95
1.83
1.01
0.98
2.00
2.23
1.95
2.08
2.29
2.24
1.86
2.88
1.92

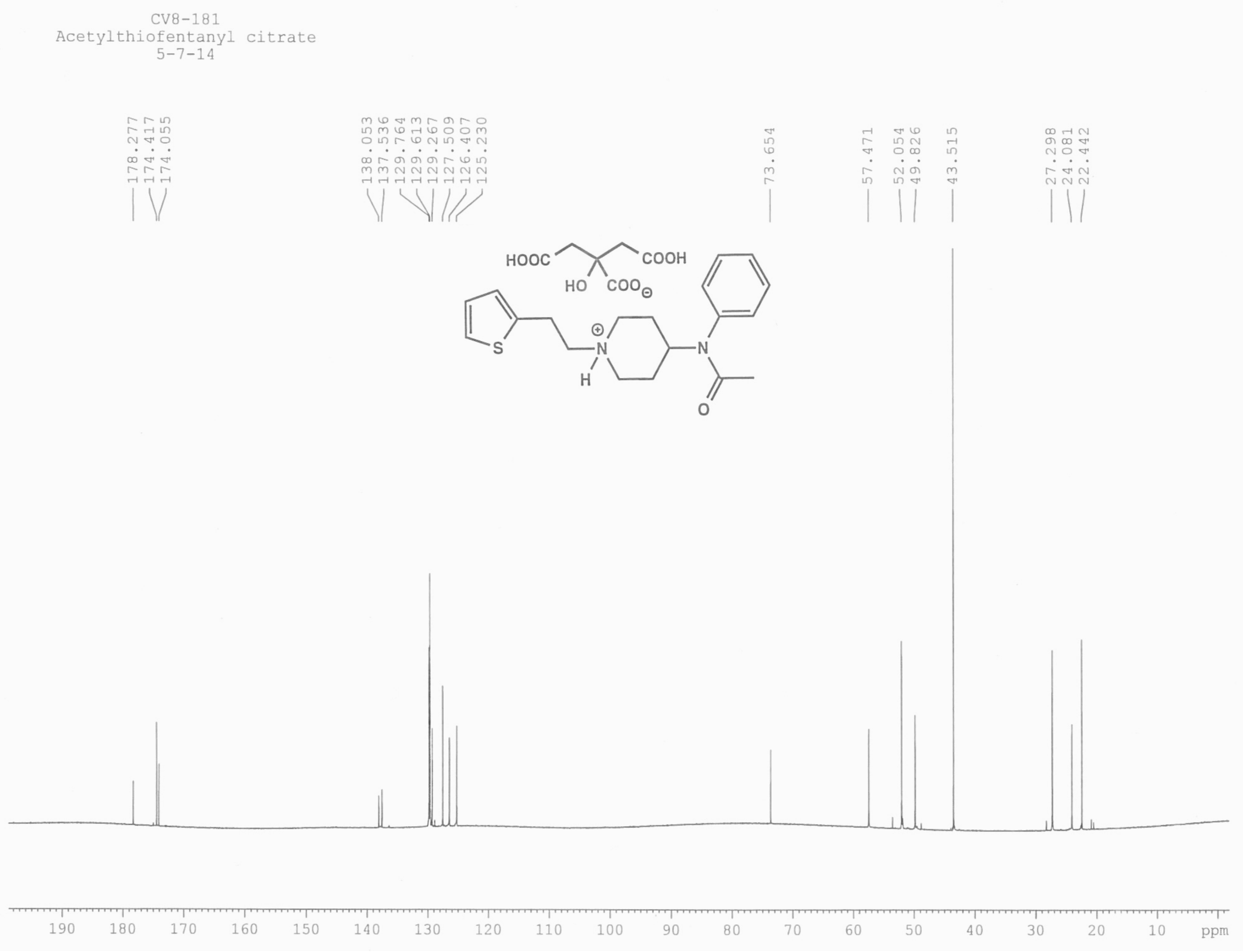
CV8-181
Acetylthiofentanyl citrate
5-7-14
178.277
174.417
174.055
138.053
137.536
129.764
129.613
129.267
127.509
126.407
125.230
73.654
57.471
52.054
49.826
43.515
27.298
24.081
22.442
HOOC
COOH
HO
COO
S
N
H
N
O
190
180
170
160
150
140
130
120
110
100
90
80
70
60
50
40
30
20
10
ppm
